# An interactive AI-driven platform for fish age reading

**DOI:** 10.1371/journal.pone.0313934

**Published:** 2024-11-18

**Authors:** Arjay Cayetano, Christoph Stransky, Andreas Birk, Thomas Brey

**Affiliations:** 1 Thünen Institute of Sea Fisheries, Bremerhaven, Germany; 2 School of Science and Engineering, Constructor University, Bremen, Germany; 3 Faculty of Biology and Chemistry, University of Bremen, Bremen, Germany; COISPA Tecnologia & Ricerca - Stazione Sperimentale per lo Studio delle Risorse del Mare, ITALY

## Abstract

Fish age is an important biological variable required as part of routine stock assessment and analysis of fish population dynamics. Age estimates are traditionally obtained by human experts from the count of ring-like patterns along calcified structures such as otoliths. To automate the process and minimize human bias, modern methods have been designed utilizing the advances in the field of artificial intelligence (AI). While many AI-based methods have been shown to attain satisfactory accuracy, there are concerns regarding the lack of explainability of some early implementations. Consequently, new explainable AI-based approaches based on U-Net and Mask R-CNN have been recently published having direct compatibility with traditional ring counting procedures. Here we further extend this endeavor by creating an interactive website housing these explainable AI methods allowing age readers to be directly involved in the AI training and development. An important aspect of the platform presented in this article is that it allows the additional use of different advanced concepts of Machine Learning (ML) such as transfer learning, ensemble learning and continual learning, which are all shown to be effective in this study.

## Introduction

Computer-assisted annotation of image data is a standard tool in marine biology since quite a while [1] with increasing amounts of automated processing over the years [2]. The progress in Artificial Intelligence (AI), or more precisely in Machine Learning (ML) in the form of deep neural networks [3] has provided a further boost to this trend [4–6]. This also holds for the application area which motivates the work presented in this article, namely the field of fish age reading.

Stock assessments rely heavily on fish age data which is primarily derived from the so-called otoliths or ear stones. These are calcified structures that form ring-like patterns influenced by the changing seasons [7] similar to the pattern of ring formation found in tree trunks as used for dendrochronological studies [8]. Hence, in the same manner, age readers count the number of such otolith rings (annuli) in order to derive the fish age estimates [9].

Often, otolith rings are not straightforward to identify or to detect. Consequently, human readers need to undergo extensive training and even attend a number of workshops with other age readers with the goal of standardizing and minimizing the subjective aspects of the process. As part of these workshops, inter-reader agreements are measured by allowing them to read a selected set of otolith images. Based on these workshops [10, 11], it was observed that there can be a concerning degree of disagreement among age estimates even for otoliths with simple patterns such as those of cod. It is therefore important to find a definitive and unbiased solution to this problem in order to prevent miscalculations involving such important biological parameter.

Hence, there has been a growing interest in recent years regarding the use of AI in age reading. Some early attempts in this endeavor dates back from the years where classical machine learning algorithms were popular along with the practice of feature engineering. These include the works of Fablet and Le Josse [12] as well as from Bermejo et al. [13] where classical neural networks and support vector machines were explored.

With the progress in deep learning, the number of studies applying AI-based methods for otolith age reading increased substantially. A lot of these studies made use of Convolutional Neural Networks (CNN) with either classification or regression formulation [14–18]. Recently, a new batch of approaches emerged using more recent concepts such as Transformers [19] and Ensemble Learning [20], indicating the continued pursuit to further improve AI-based approaches for otolith age reading.

There are some concerns, however, when it comes to the black-box nature of many of these implementations. This is primarily due to their lack of compatibility with the traditional manual methodology in which the rings are explicitly counted to derive the age values. Hence, newer designs were recently developed with the goal of making the process as compatible as possible with the ring-counting procedure. Bojesen et al. [21] applied a novel method involving generative models to specifically mimic the ring annotations often made by readers especially during workshops serving as visual guides for easier ring identification. That is, certain markings (dots) are placed by the AI-method within the image indicating the ring portions it detected. Likewise, in own work, Cayetano et al. [22] successfully created automated ring annotations using Mask R-CNN [23] and U-Net [24], which are popular methods for object detection and segmentation. These methods are shown to satisfactorily create masks of the rings at certain reading axes from which age estimates are derived through a simple automated counting procedure. In this way, a human can easily comprehend the way the fish age was determined and validate the result if needed.

To further increase the acceptance and trust in AI-based approaches for age reading, we extend the use of explainable methods by creating a platform which enables the readers to directly participate in the development and training of such AI models, similar to the idea behind DeepOtolith [25]. In this study, we developed an interactive web-based application that houses both Mask R-CNN and U-Net algorithms and makes them accessible for age readers via an intuitive user interface. An important scientific aspect introduced in this work is that this approach allows using additional, more advanced Machine Learning concepts, which boost the performance as shown in this article.

From the perspective of an AI-based fish-age-reading tool, the idea is to provide a mixed collection of AI models composed of user-trained models, along with the models we developed [22] as well as those generic models trained from common objects such as VGG [26] and Mrcnn-Coco [27, 28]. This opens up additional options to use advanced ML concepts to further improve the performance. Concretely, the following three contributions are presented in this article. First, transfer learning is incorporated where existing models are reused to initiate a new round of training on a new set of otolith images. Second, multiple models can be consolidated during the testing/prediction stage using an ensemble method to create an aggregate prediction, which is significantly better than the individual model predictions. Third, continual learning is used, i.e., the user can build an initial model from a specific dataset which will be trained repeatedly each time a new dataset comes without completely forgetting the original dataset it was trained on.

## Materials and methods

### Implementation and design

We provide here a short overview of the technical details of the platform as background information. This also includes information on the basic ML methods that we build upon before the main conceptual contributions of this article are presented in the succeeding sections.

The platform is implemented as a web application which is currently hosted at Thünen Institute on a Linux server with high-performance graphics cards for deep learning and using a docker setup for Tensorflow-GPU/Keras library [29, 30]. Currently, the website access is limited within the Thünen local network but a portable standalone version (Windows only) can be downloaded for testing, exploring and trying out some selected useful features. The implementation (DOI 10.5281/zenodo.8341297) is based on Python and the Django web framework [31]. The portable version (DOI 10.5281/zenodo.10954470) is packaged in a zip file, containing all the necessary libraries for starting the webserver and can even be configured to allow other local machines to connect.

Similar to the previous work [22], preliminary image processing steps were included such as outer contour detection and selected image adjustments. For instance, the watershed algorithm [32] can be employed for automatically segmenting the otoliths from its background. In case of errors in the contour detection, an annotation toolkit is integrated for the end-user, namely VIA or Visual Geometry Group Image Annotation tool [33].

Likewise, the web application also provides the two deep learning methods for fish age reading that we previously developed [22], which are based on Mask R-CNN [23] and U-Net [24]. These two methods were demonstrated to be accurate and robust in estimating the fish age while at the same time, creating image annotations of the otolith rings (i.e., image masks or markings showing the identified otolith annuli). Both algorithms are supervised methods requiring ground truth labels. The labels are in the form of mask annotations within an image indicating the region of interest to be detected or segmented, respectively, by Mask R-CNN and U-Net.

**Fig 1:**
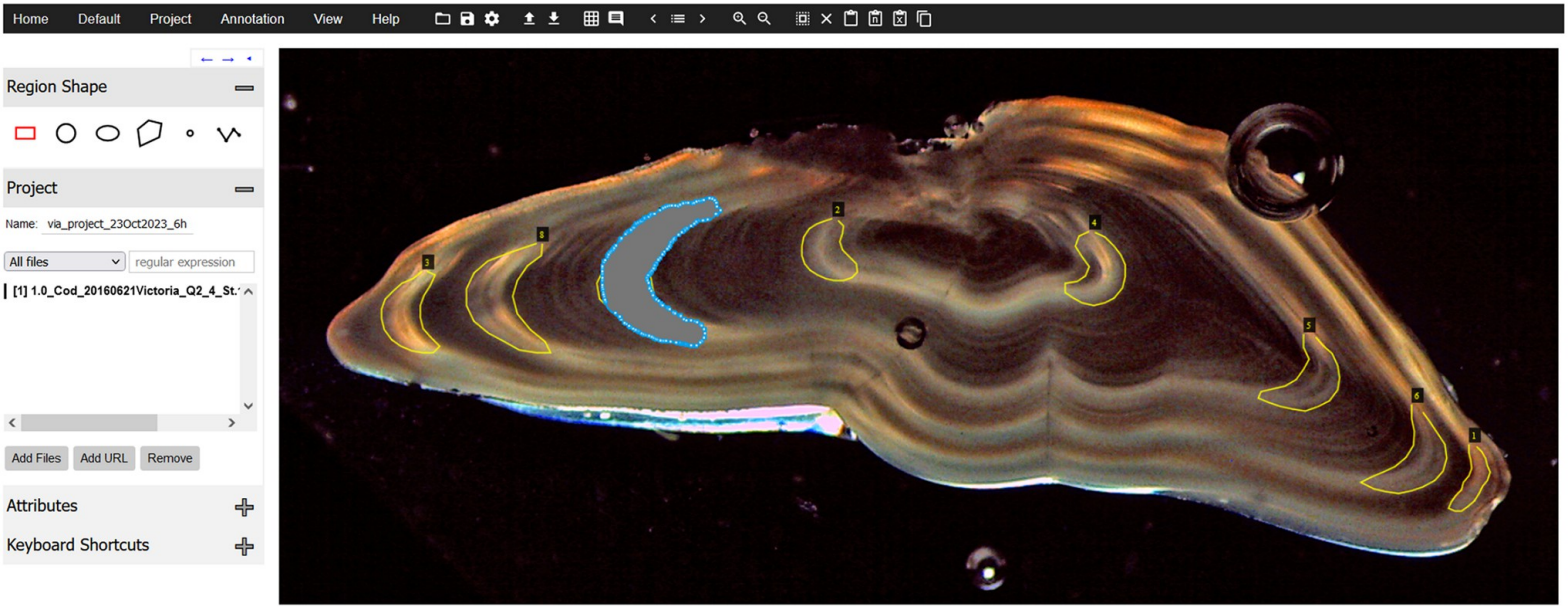
Improved VIA annotation toolset. Sample annotation accomplished using the brush tool we implemented within the integrated VIA toolkit.

As mentioned before, VIA is integrated, which can be used to create ground-truth annotations using its drawing tool sets. Furthermore, we augmented VIA and integrated our own custom brush tool, which makes the creation of annotations for this specific application case faster and easier. Fig 1 shows an example of an irregular shape created using the brush tool which will be otherwise tedious to draw using the default VIA drawing toolkits.

**Fig 2:**
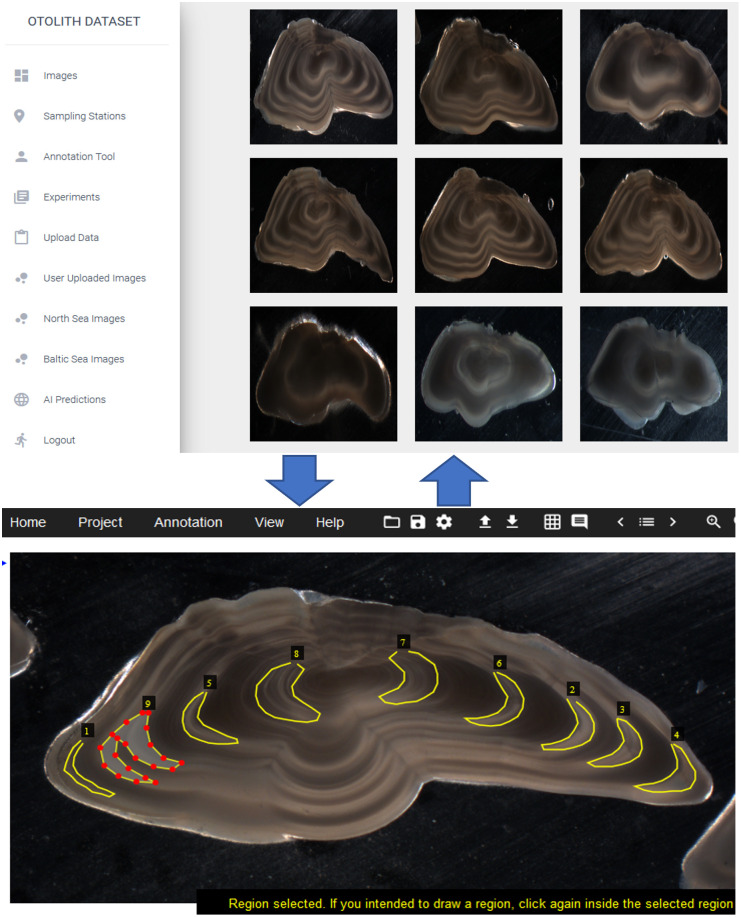
With AI assistance. The AI-assisted annotation we implemented where the users can edit the initial annotation given by the AI which can be saved and utilized in the platform.

As another modification of the VIA tool to ease the annotation process, we also utilize existing pretrained otolith models from own work [22] that can perform reasonable initial annotations on new sets of images to be edited/corrected by end-users as necessary. That is, this feature serves as an AI-assistant that helps the users create the ground-truth labels for new images they upload. The process is illustrated in Fig 2.

### Experiments and data partitioning

**Fig 3:**
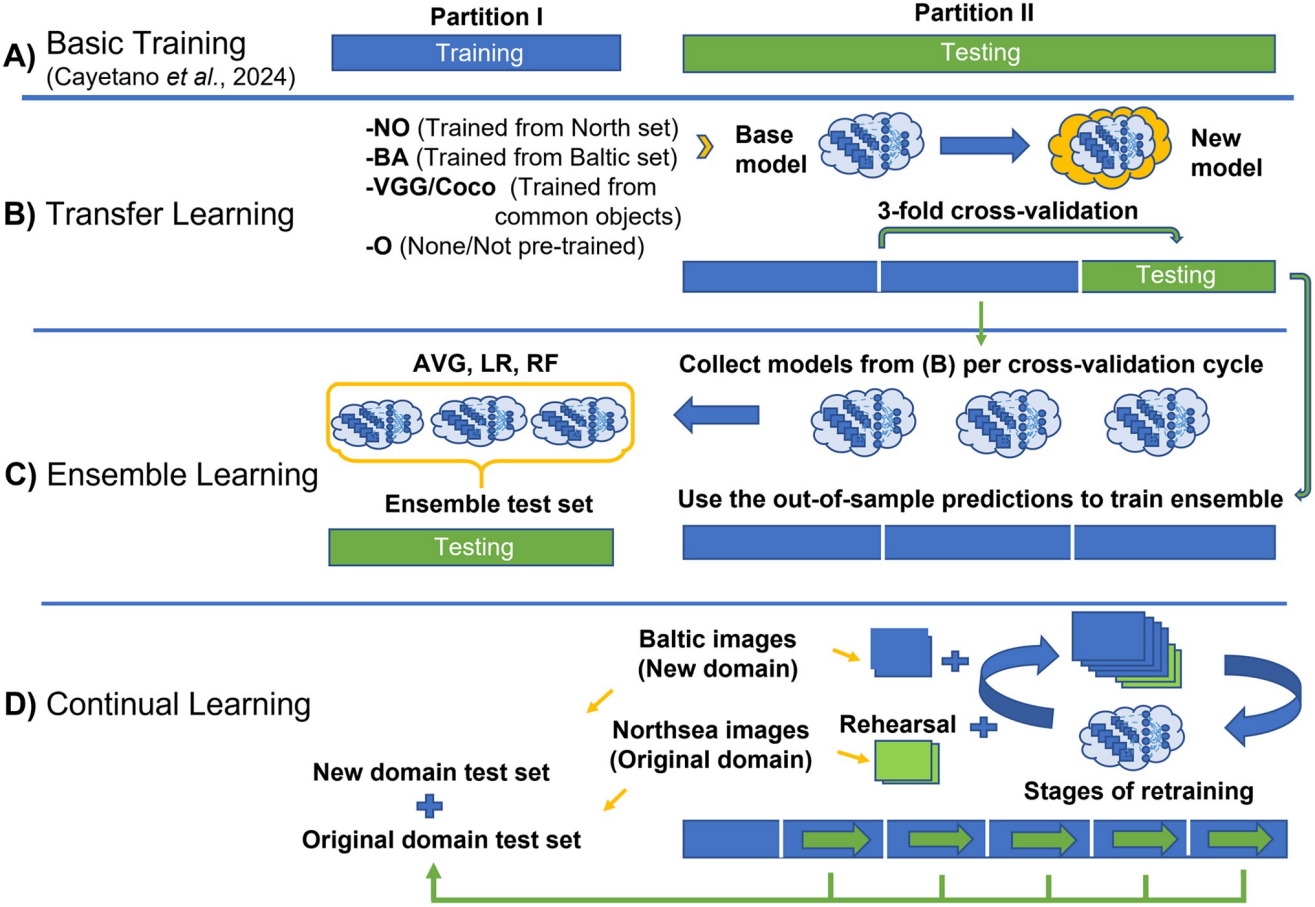
Training-testing splits. The partitioning of the dataset for the different experiments we conducted namely (A) basic training done from the previous study (B) transfer learning (C) ensemble learning (D) continual learning. The training set is marked in blue while the test set is marked in green. In addition, the green colored arrows point to the previous test sets that will eventually become training sets for the succeeding stages of the relevant experiments.

After creating the annotations, the next step is to conduct various experiments to study the advanced AI techniques namely transfer learning, ensemble learning and continual learning. First, the validation data needs to be created which will be used for measuring the performance of the model during training so that the best-so-far state can be saved and retrieved accordingly. In this study, the validation data was derived simply by using horizontal flipping of the training data. The test data, however, will be different for each set of training and testing experiment we conduct which is illustrated in Fig 3 and summarized in Table 1.

**Table 1:** The different experiments conducted along with the details on how each experiment utilized the partitions created for the two datasets. It should be noted that in the previous study [22], the Partition I was used for training while Partition II was used for testing.

Experiments	Partition I	Partition II
**Transfer Learning**		
Training	No	Yes (cross-validation)
Testing	No	Yes (cross-validation)
**Ensemble Learning**		
Constituent training	No	Yes (excluded some pretrained weights)
Constituent testing	Yes	No
Ensemble training	No	Yes (out-of-sample predictions)
Ensemble testing	Yes	No
**Continual Learning**		
Training	No	Yes (stage-by-stage training)
Testing	No	Yes (test on incoming/next stage)

The main idea behind the data partitioning is to have two major subsets namely Partition I and Partition II. Partition I is the smaller age-balanced partition for both datasets (20% for North Sea dataset and around 13% for Baltic dataset) while Partition II is composed of the larger remaining set of images. In our previous paper [22], Partition I was used for training while Partition II was used for testing as illustrated in Fig 3A. In contrast, in this new study, Partition II was used primarily for training while the testing involves varying configurations depending on the purpose which are summarized in Fig 3B–3D and in Table 1. Also, for additional details regarding the datasets, supplementary materials are available namely S1 and S2 Tables.

### Transfer learning

In this experiement, there are two types of training configuration explored which can either be from scratch or from reusing previous models. The former trains a model from the ground up using the default random weight initializations of the CNN. The latter takes advantage of transfer learning to load existing model weights during network initialization from which the training can continue.

There are essentially three ways by which the user can select existing models for transfer learning. First is the basic case involving existing models published by the AI community which are trained from common objects such as VGG [26] and Mrcnn-Coco [27, 28]. The second case involves reusing models trained from application specific data, here otolith images, such as the ones we already provided [22] which can be reused for any otolith image dataset even with different species or domains. The last case is an extension of the second case and it involves the use of highly domain-specific models, i.e., training involves images with the same application specific data and the same characteristics (e.g. lighting, orientation), which hence potentially requires only a simple model update.

To fully utilize our image collection, we have to reuse the larger subset (i.e., the Partition II shown in Fig 3) in order to conduct new rounds of training with the different scenarios mentioned in this study. To evaluate the use of transfer learning, we conduct a 3-fold cross-validation experiment (Fig 3B) where this subset is divided into three parts. Then, following the usual procedure, each round of cross-validation uses one of the three folds as test set from which the performance of the scenarios can be evaluated.

### Ensemble learning

In this section, we present the options for performing predictions on a separate set of images. In the basic case, the trained model created by the user is tested individually on a different set of images prepared for testing. The application’s output are the predicted annotations within the image itself along with the estimate of the age value. In the more advanced scenario, the predictions of the models can be combined to create one aggregate prediction using the so-called ensemble approach.

Ensemble Learning is a machine learning technique in which multiple models are combined into a single aggregate model having a prediction which is computed or even learned from the individual predictions of its constituents [34]. For simplicity, we can describe it as a form of meta-analysis or meta-learning where patterns are derived from the individual model predictions. For this experiment, the constituent models we used are the models trained in the previous experiment involving transfer learning. The following three types are considered here:

- **Model Averaging**
  This is the simplest method for creating an ensemble model. It only involves simple averaging of the predictions of the individual models. As reviewed by Ganaie et al. [34], there are cases in which this can be a reasonable choice compared to some other more complex ensemble approaches.
- **Linear Regression**
  In this method, an additional round of training is required using linear regression as the meta-learner and using the deep learning model predictions as the inputs. As illustrated in Fig 3C, this training is performed on the out-of-sample predictions from the 3-fold cross-validation conducted in the transfer learning experiment. Then, the testing of the ensemble can be performed on the original training set from the previous study [22], which we now designate as the test set. This means, however, that for this experiment, we have to exclude the use of some transfer learning models having base weights that were trained previously on this specified test set.
- **Random Forest**
  Random forest is a classical ML algorithm which by definition, also functions as an ensemble model for classification and regression [35]. For our purposes, we treat it as a typical classifier that can learn patterns from the predictions of the deep learning models. Likewise, as shown in Fig 3C, we train it from the test predictions of the individual models from the transfer learning experiment and test it on the original training images used in our previous study [22]. Hence, we will also exclude any model containing pretrained weights that has been derived from this currently designated test set.

### Continual learning

In this section, we present the use of continual learning. As mentioned in the previous section on AI-assisted annotation, this is a more advanced feature that can be used for creating ground-truth annotations where the user can choose to retrain the existing model that performs the initial annotation with the new annotated images assisted by the model itself.

**Fig 4:**
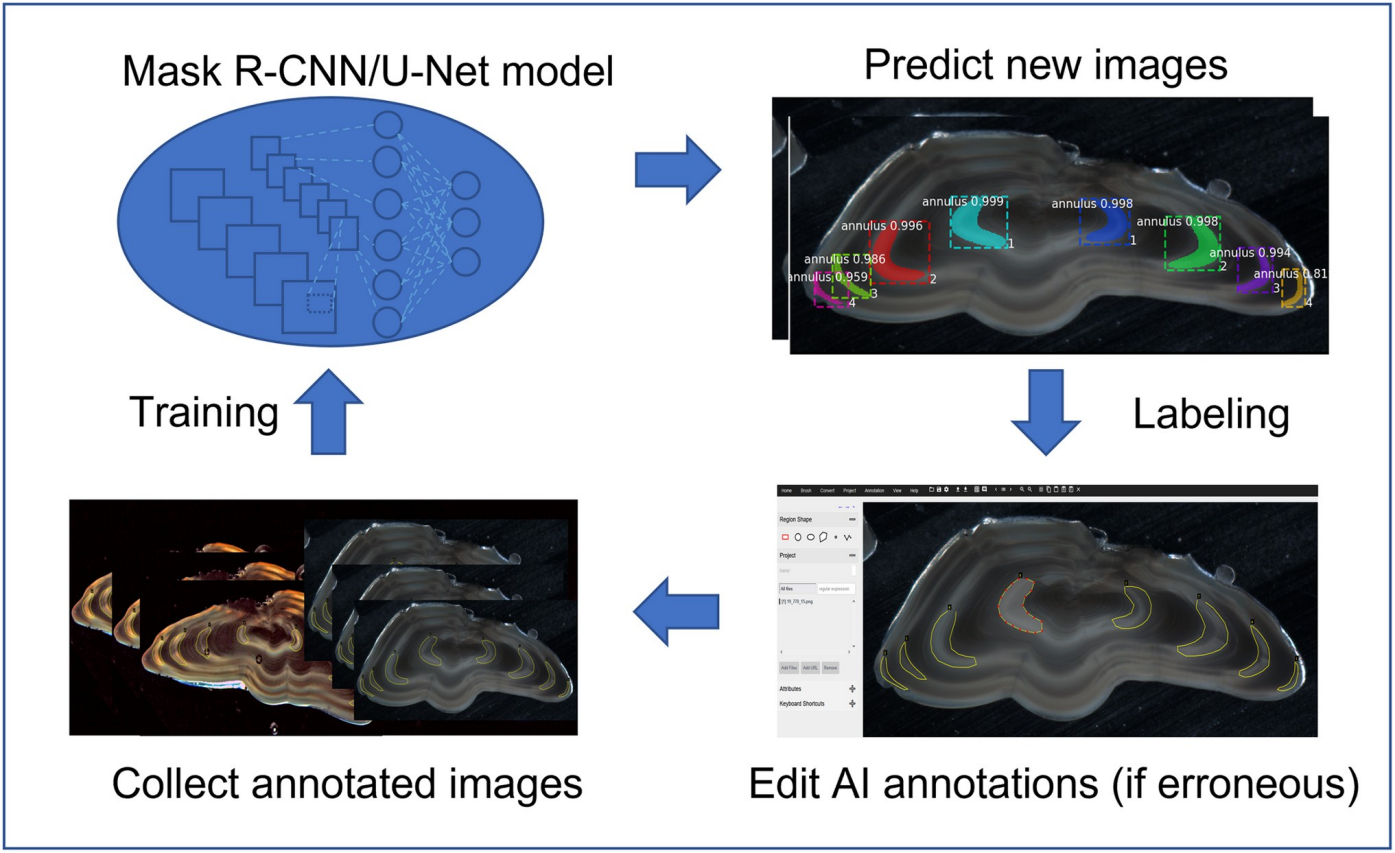
Continual learning workflow. The self-sustaining loop for continual learning where the model predictions on a new dataset can be edited, corrected and approved by the user so that it can be used to conduct retraining to utilize the new labeled images.

In this functionality, users will be able to perform training in batches of data and with each batch, the model from the previous batch will be reloaded to annotate the new batch (with corrections from the user if necessary) and then subsequently, conduct training using the same set. This workflow loop is summarized in Fig 4.

The main hurdle, however, is the so-called catastrophic forgetting or catastrophic interference [36, 37] where the model will eventually not recognize the previous dataset it has learned while it is currently being trained on a completely new dataset. Here, we investigate this phenomenon by observing the deterioration of prediction on the original dataset or source domain (North Sea dataset, in this case), as the model undergoes retraining using different training sets containing images from a new domain (i.e., Baltic Sea dataset). In addition, we explore the commonly proposed solution for catastrophic forgetting which involves rehearsal [38, 39]. We employ the basic rehearsal method in which we include some images from the original domain and mix it on each new batch of images from the new domain to be used for further training. In this manner, the model has the chance to “review” or “rehearse” its original domain while also learning from the new set.

## Results and discussion

**Fig 5:**
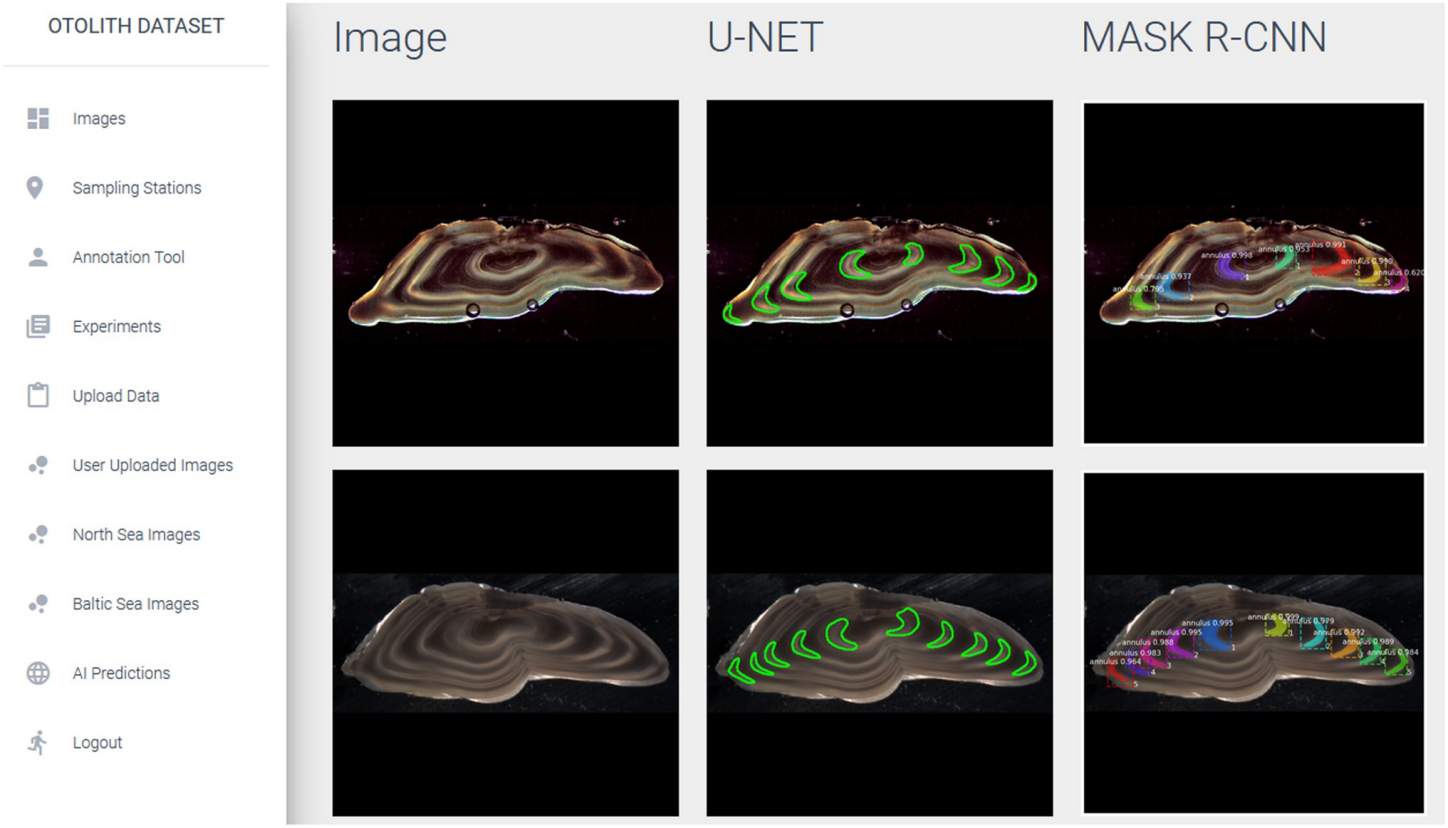
Webpage showing predictions. Automated image annotations created by the two different AI methods, U-Net and Mask R-CNN, as shown on the web application.

Fig 5 shows the website page containing a sample set of images with the corresponding predictions and annotations of the AI models that can be created using the web application. As shown in the figure, color markings of otolith rings are directly placed within the image making it easy to interpret and verify by the age reader.

### Transfer learning

**Fig 6:**
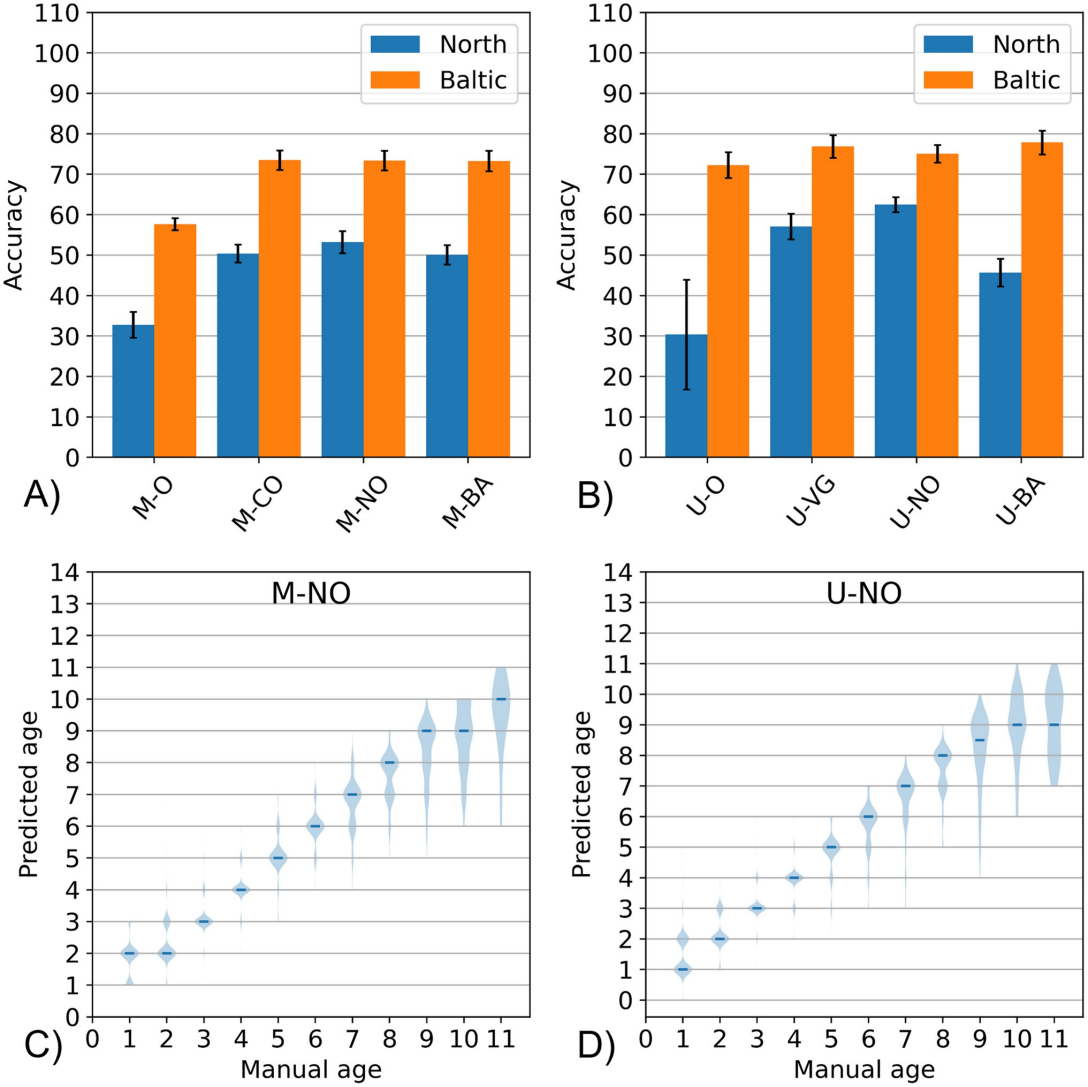
Cross-validation performance. The performance of the different models tested using 3-fold cross-validation (3 repeats using 3 different otolith base weights creating a total of 9 runs). All Mask R-CNN models start with “M” while all U-Net models start with “U”. The models tagged with “-O” refer to those without transfer learning while the rest indicates the identifier of the pre-existing base models (-CO refers to the Coco model, -NO refers to the North Sea-trained model, -BA refers to the Baltic-trained models, and -VG refers to the VGG model).

In Fig 6, the result of the experiments with the different types of transfer learning is shown. The evaluation of performance was conducted via 3-fold cross-validation as described in the Methods section.

It can be seen that for both algorithms (Mask R-CNN and U-Net), the model without any type of transfer learning has consistently attained the least accuracy. In addition, it can be seen that, in the case of the North Sea dataset, the model with pretrained weights based on North Sea images attained the highest accuracy for both Mask R-CNN (53.2%) and U-Net (62.5%). In the case of the Baltic Sea dataset, the model with pretrained weights based on Baltic Sea images also performed outstandingly attaining the second highest accuracy (73.3%) for Mask R-CNN and the highest accuracy (77.8%) for U-Net.

In addition, we further elaborate the performance of the best models created with transfer learning to see the distribution of their predictions on each age classes as shown in Fig 6(C) and 6(D). It can be seen from the violin plot, that the predictions of the best models are excellent from ages 1–8 and start to decline from ages 9 onwards. Nevertheless, the overall performance can still be considered satisfactory despite using an imbalanced training set, which is a consequence of cross-validation, in contrast to the one used in the previous study [22] where each age group contains the same number of images (random sampling with rebalancing).

With these results, the advantage of transfer learning has been demonstrated and shown to be superior compared to training from scratch without any pretrained weights. This aligns with the results of various AI-based age reading studies such as the works of Moen et al. [14], Martinsen et al. [17] and Ordoñez et al. [15], where they previously demonstrated the effectiveness of pretrained Inception, Xception and VGG, respectively. In addition, our results also indicate that, in some cases, the use of base models pretrained from otolith images obtains better performance compared to using typical generic models pretrained from common objects. Hence, in this study, we present another future perspective to obtain further improvements by encouraging the development of more otolith base models that can be reused by the community using the concept of transfer learning.

### Ensemble learning

As mentioned, the ensemble approach is a meta-learning process requiring the model predictions as inputs for training and testing. Hence, we require sufficiently diverse models that can be combined accordingly. For this, we have reused the models trained using the transfer learning experiment from the previous section.

**Fig 7:**
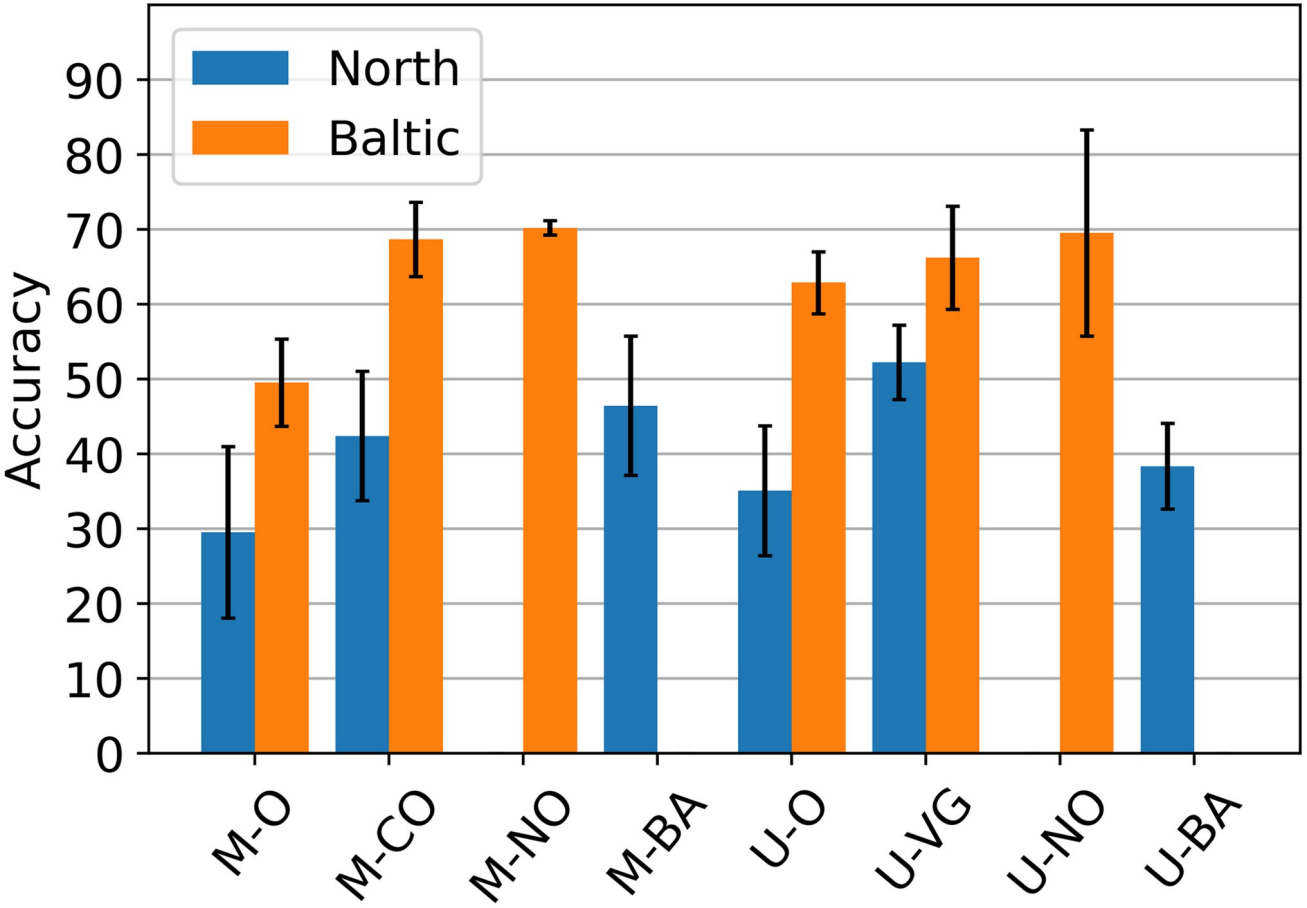
Performance of constituent models. Performance of each individual model used to create the ensemble models (n = 3). In compliance with standard practice, all models from the cross-validation experiments are discarded. To measure the performance of the methods, i.e., those starting with M- represents Mask R-CNN and U- for U-Net, a final training was done where all images from the three folds of cross-validation were included. Then, evaluation was done on an unused set, i.e., the set which was used for training in the previous study [22] which is now designated as the test set.

The training set of the ensemble would be the predictions of each individual model on the out-of-sample test set created via 3-fold cross-validation from the previous section. Then, for testing and proper analysis, we need to evaluate the performance of the ensemble on the training set used in the previously published study [22], which is now currently designated as the test set. In order for this to work, however, it was necessary to exclude the models containing weights trained from that set because they will naturally be familiar with it, making the results too optimistic. Hence, in Fig 7, it can be seen that we evaluate six models per dataset instead of eight as done in the previous section. The accuracy values for these selected models are plotted in a bar chart which we will use to compare with the combined/ensemble models.

**Fig 8:**
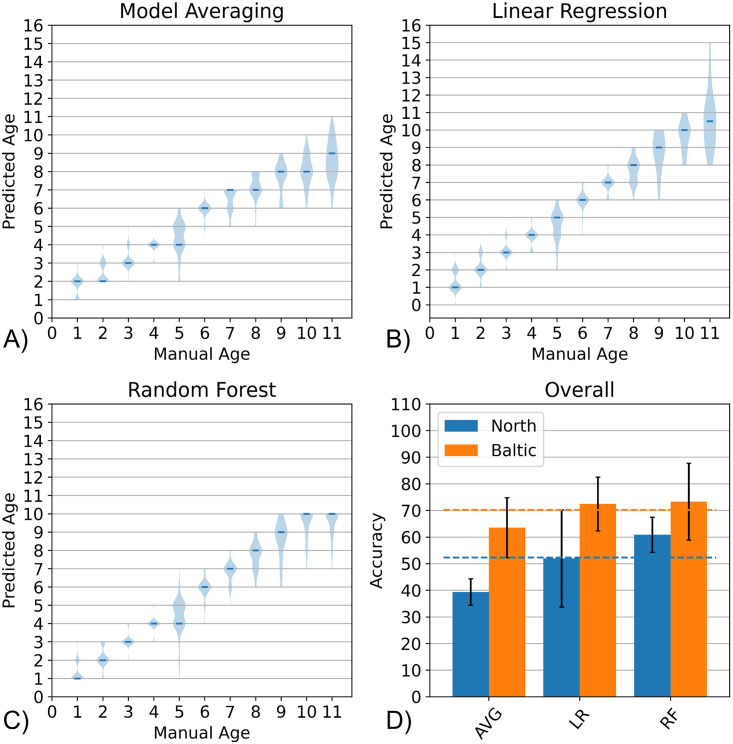
Ensemble performance. Distribution of age predictions (n = 3) using the three ensemble methods namely (A) Model Averaging (AVG) (B) Linear Regression (LR) (C) Random Forest (RF). The overall accuracy of the three methods is summarized in a bar chart (D) along with the accuracy of the best constituent model indicated by dashed lines as obtained from the tests for constituent models (Fig 7).

In Fig 8, the three types of ensemble approaches are evaluated. Fig 8(A)–8(C) show the distribution of predictions on each age classes produced by the three ensemble methods. It can be observed that the ensemble using Model Averaging produced the worst distribution among the three methods. When it comes to the distribution across all age groups, the Linear Regression produced the best trend where the age-wise median values were located on the expected position with the exception of age 11. For the Random Forest method, both age 5 and age 11 otolith images were heavily under-estimated but the rest of the age groups are excellently predicted. To know which one is superior, we also plotted their corresponding accuracy values (Fig 8D). It can be seen that Random Forest (RF) attains the highest accuracy values for both North Sea and Baltic Sea images. Moreover, for both datasets, it surpasses the performance of its best constituent model which is depicted in the plot as dashed blue and orange lines, respectively. This means that the use of an ensemble, particularly in form of a Random Forest, can be used to further improve predictions. These results complement the findings from Moen et al. [20], where they showed how simple averaging ensemble already provides a decent increase in accuracy. In our study, we explored additional ensemble types apart from model averaging and showed that further improvements can be obtained with other more sophisticated ensemble methods.

### Continual learning

**Fig 9:**
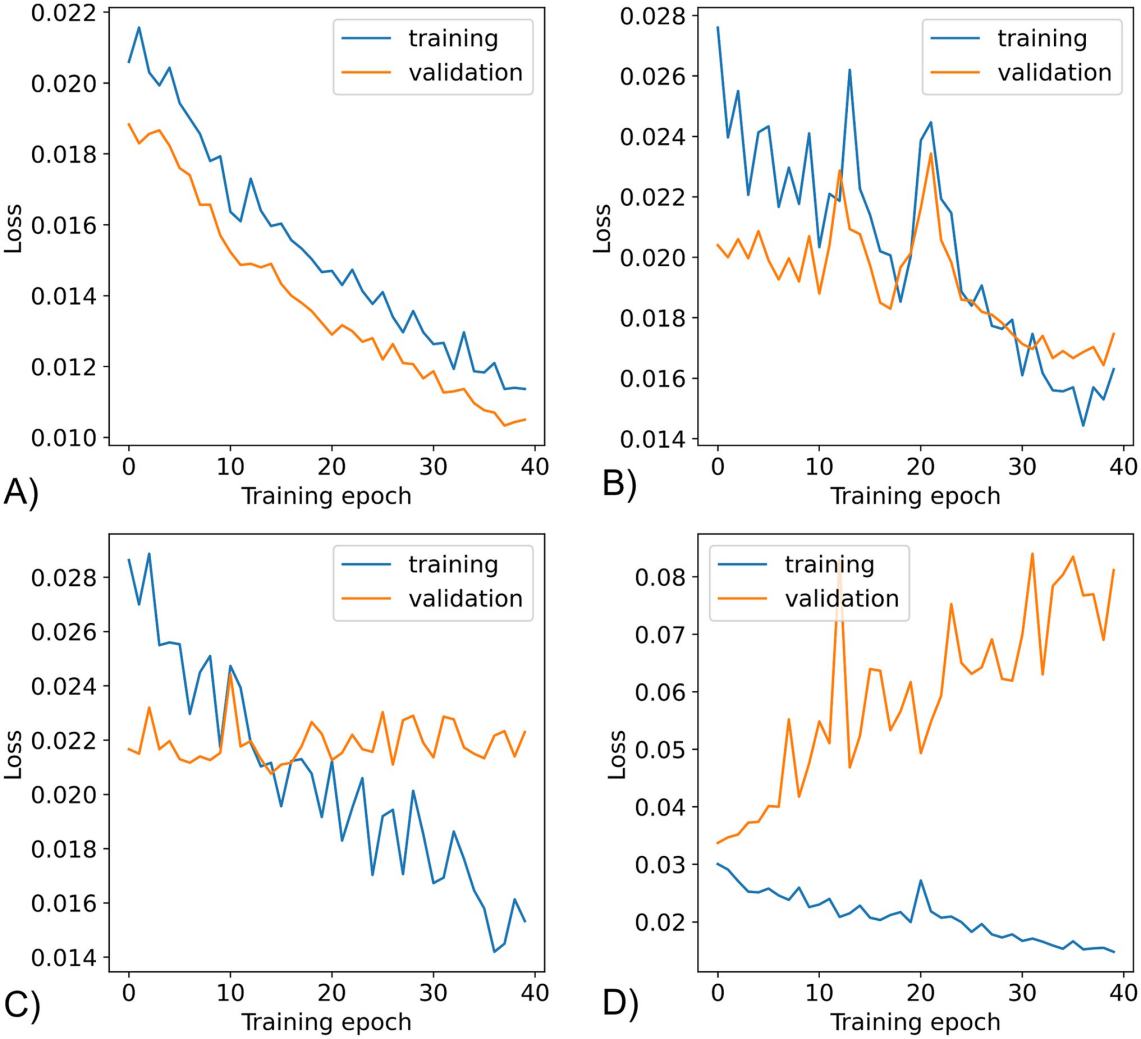
Catastrophic forgetting. The effect of catastrophic forgetting investigated (n = 3) using four different training scenarios involving images from original domain (North Sea) and the new domain (Baltic Sea). (A) training set with purely old domain images (B) mixed training set with 33.3% new and 66.7% old images (C) mixed set with 66.7% new and 33.3% old images (D) training set consisting purely of new domain images.

In this section, we first investigate the phenomenon known as catastrophic forgetting in which a trained model tends to forget its original training set once retrained with a completely new dataset. In Fig 9(A)–9(D), we illustrate this behavior by reusing an existing U-Net model previously trained from the North Sea images and retraining it in four different ways.

To see how well it remembers the original training set, we use a validation set composed of the exact images it was previously trained on. We then plot the training loss (blue) and the validation loss (orange) as the model undergoes retraining under four different scenarios. In Fig 9A, we run a baseline scenario where the training set is composed of the exact images from the original training without introducing new images. This represents the case where the model is still completely familiar with the validation set resulting to the expected highly optimistic loss plot. Afterwards, we perform additional experiments where we add new domain images into the training set. In Fig 9B, we create a mixed training set composed of 33.3% new images and 66.7% old images. It can be seen how the model still manages to have improvements in both training and validation loss albeit in a more unstable manner compared to the baseline scenario. Next, we increase the proportion of the new images (66.7%) for training and plot the resulting loss in Fig 9C. Here, the improvement is now heavily leaning towards the training while the validation loss is maintained (i.e., neither improves nor deteriorates). Finally, we explore the case where all training images are from the new domain. In Fig 9D, it can be seen that the validation loss gets worse while the training loss improves indicating that the model’s familiarity with the old training images deteriorates as the model learns the new domain images. This deterioration of the validation loss represents the catastrophic forgetting phenomenon as the model encounters increasing proportion of unfamiliar images during training.

In the next experiment, we investigate the most common solution proposed for handling catastrophic forgetting which is the rehearsal method [38, 39]. For this, we evaluate a scenario with and without rehearsal. This is done separately for Mask R-CNN and U-Net using pretrained weights based on North Sea images. Hence, similar to the previous experiment, we consider the North Sea dataset as the original domain while the Baltic Sea dataset serves as the new domain. For rehearsal, we use a mixed training set where the number of original domain images is 50% of the new domain images. These old images are reintroduced in each subsequent training stage as described in the Methods section (Fig 3D).

**Fig 10:**
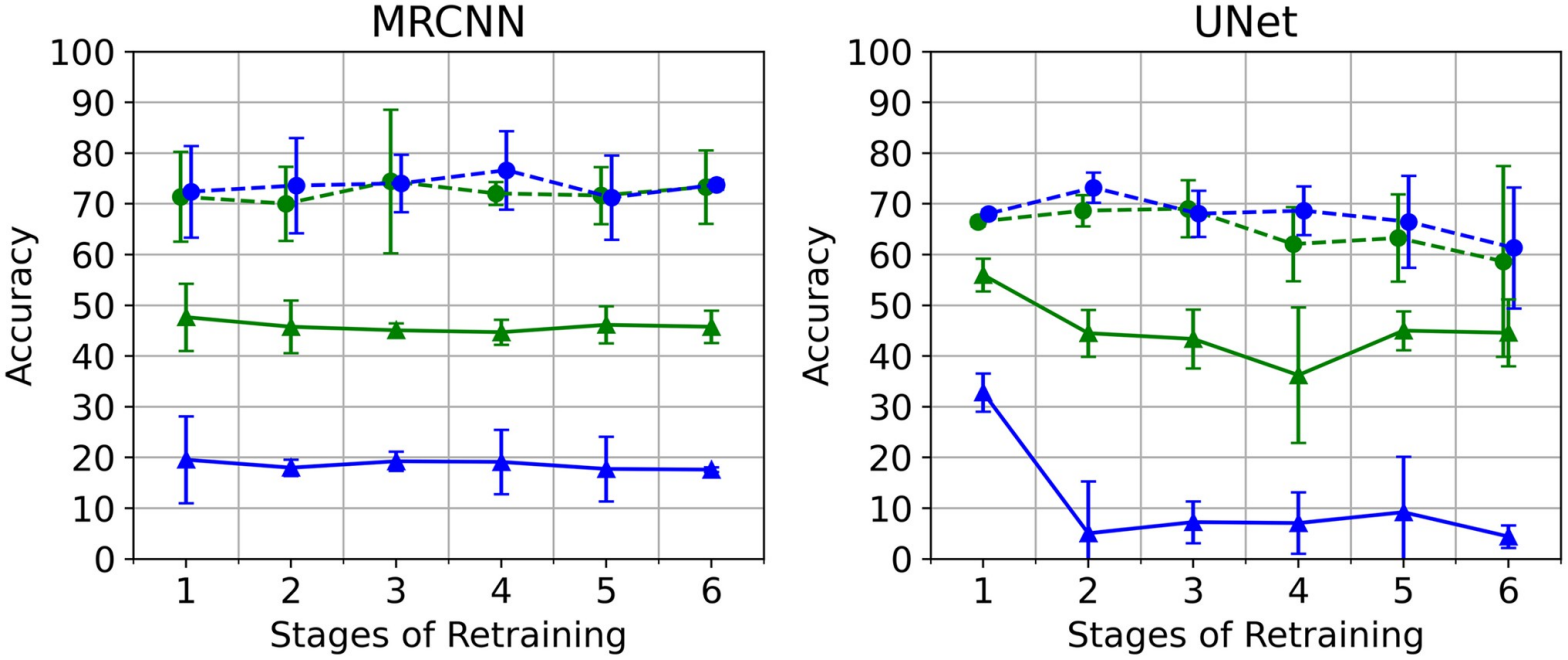
Performance with- and without rehearsal. The accuracy values for the continuously learning models at six stages of retraining. The green lines indicate the models with rehearsal while the blue lines indicate those without rehearsal (normal training). The continuous lines (with triangle markers) represent the accuracy for the original domain while the dashed lines (with circular markers) represent the accuracy for the current/new domain. In both algorithms, the models with rehearsal are able to maintain good accuracy for both the original domain and the current domain. The models without rehearsal only performed satisfactorily for the current domain and drastically became unfamiliar with the original domain it was previously trained on.

The accuracy plots in Fig 10 show the effectiveness of the rehearsal approach in preventing catastrophic forgetting. The models with rehearsal, shown in green lines, are able to maintain good accuracy values for the original domain as well as for the new domain that they are currently learning from. This observation is true for both Mask R-CNN and U-Net. The models without rehearsal are only good for the current domain and they become completely unfamiliar with the original domain they were previously trained on. Similar to the previous experiment, the performance of the models on the original domain deteriorates drastically as they learn from the new domain without rehearsing on the old images.

**Fig 11:**
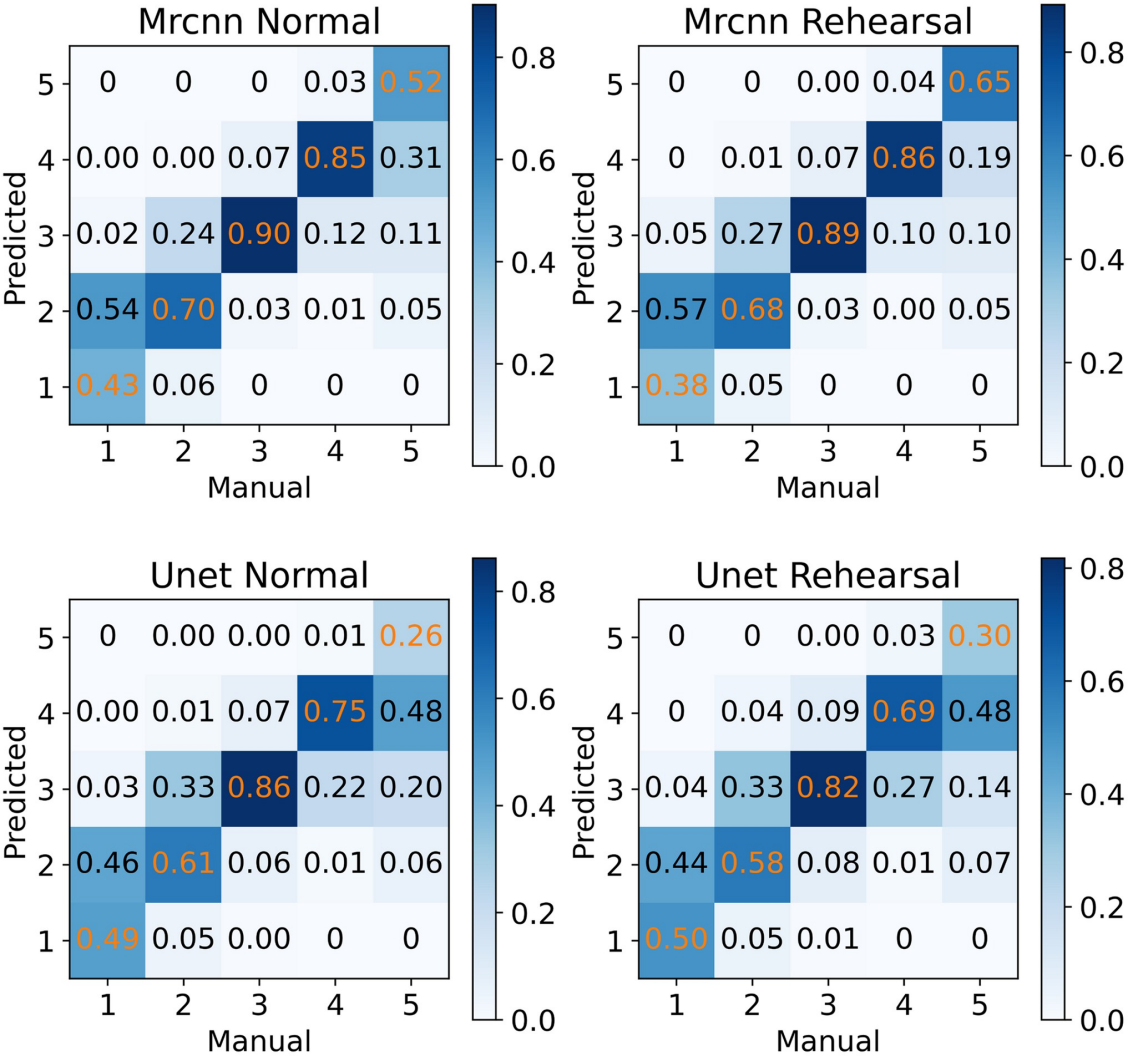
Age-wise accuracy. Confusion matrices containing the age-wise accuracy values of the models without rehearsal (Normal) and with rehearsal (Rehearsal) when evaluated on the current domain. Despite the inclusion of old images from the original domain via rehearsal, the performances of the rehearsal models are not affected for the new domain; this is indicated by the similar accuracy values attained by both model variants. The negligible percentage of predictions outside age range 1–5 are not shown.

As a final comparison, we present confusion matrices showing the prediction accuracy of the models with- and without rehearsal evaluated on the current domain. As seen from Fig 11, all models have comparable performance, i.e., they are all excellent in predicting ages 2–4 but performed slightly worse for ages 1 and 5. For each algorithm, the two variations of training (with- and without rehearsal) performed similarly as seen by the age-wise accuracy values along the diagonal of the corresponding matrix. This observation indicates that with rehearsal, the performance of the models on the new domain remains unaffected even when the training is conducted on a mixed set composed of both the old and the new images.

## Conclusions

We presented work on using higher-level Machine Learning strategies in the context of automated fish age reading based on images of otoliths. This is a highly relevant scientific and economical problem as fish age is an important biological variable required as part of routine stock assessment and analysis of fish population dynamics.

The presented work is grounded in an easy to use, web-based tool-chain targeted at human age-readers as end-users without a background in Artificial Intelligence. The tool-chain provides methods the end-users are already familiar with, e.g., standard image processing and annotation tools, but also explainable AI in form of ring segmentations and automated counting.

An important aspect of the presented approach is that higher-level ML concepts such as transfer learning, ensemble learning and continual learning can be employed. It has been shown in this article that these techniques lead to improved performance which can be highly beneficial and timely especially with the recent growing interest in the use of AI for fish age reading. First, it was demonstrated here how transfer learning increased the overall accuracy especially when otolith base models are used instead of the generic base models trained from common objects. Second, it has been shown that, with ensemble learning especially using a Random Forest meta-learner, there is a further improvement in accuracy compared to just the individual performance of the constituent models. Lastly, using continual learning coupled with a basic rehearsal approach, it has been shown that one can create a single model which has an excellent accuracy for different datasets (or domains) making it unnecessary to train separate models for each set. In this study, not only that these concepts were explored in detail but also, they were packaged into a web-based platform where they can be easily utilized by end-users, even those without coding or programming background.

## Supporting information

S1 Table The number of images per age class and within each partition for both datasets. The Partition I is the smaller partition but contains the same number of images for all age classes. The Partition II is the larger partition having imbalanced number of images.
(PDF)

S2 Table The number of images per species used in the study. The North Sea dataset is composed mainly of saithe images while the Baltic dataset consists of purely cod images.
(PDF)

S1 File (ZIP)
