# Supplementary material for: An interactive AI-driven platform for fish age reading
Source: PLoS One. 2024 Nov 18;19(11):e0313934. doi: 10.1371/journal.pone.0313934 (PMC11573220; doi:10.1371/journal.pone.0313934)
Supplement: S1 Table — The Partition I is the smaller partition but contains the same number of images for all age classes. The Partition II is the larger partition having imbalanced number of images. (PDF) [file pone.0313934.s001.pdf]

Table S1. The number of images per age class and within each partition for both datasets. The Partition I is the smaller partition but contains the same number of images for all age classes. The Partition II is the larger partition having imbalanced number of images.

**Age Group**	**Partition I**	**Partition II**	**Total**
**North Sea Dataset**			
Age 1	12	26	38
Age 2	12	37	49
Age 3	12	56	68
Age 4	12	63	75
Age 5	12	74	86
Age 6	12	58	70
Age 7	12	53	65
Age 8	12	57	69
Age 9	12	47	59
Age 10	12	41	53
Age 11	12	16	28
**Baltic Dataset**			
Age 1	30	196	226
Age 2	30	247	277
Age 3	30	313	343
Age 4	30	237	267
Age 5	30	12	42
