# Supplementary material for: An interactive AI-driven platform for fish age reading
Source: PLoS One. 2024 Nov 18;19(11):e0313934. doi: 10.1371/journal.pone.0313934 (PMC11573220; doi:10.1371/journal.pone.0313934)
Supplement: S2 Table — The North Sea dataset is composed mainly of saithe images while the Baltic dataset consists of purely cod images. (PDF) [file pone.0313934.s002.pdf]

Table S2. The number of images per species used in the study. The North Sea dataset is composed mainly of saithe images while the Baltic dataset consists of purely cod images.

**Species**	**North Sea**	**Baltic Sea**	**Total**
Gadus morhua (cod)	194	1155	1349
Pollachius virens (saithe)	351	-	351
Melanogrammus aeglefinus (haddock)	78	-	78
Merlangius merlangus (whiting)	37	-	37
