# Supplementary material for: An interactive AI-driven platform for fish age reading
Source: PLoS One. 2024 Nov 18;19(11):e0313934. doi: 10.1371/journal.pone.0313934 (PMC11573220; doi:10.1371/journal.pone.0313934)
Supplement: S1 File — (ZIP) [file pone.0313934.s003.zip › USER_MANUAL/UserManual_AdvancedAI.pdf]

Transfer Learning

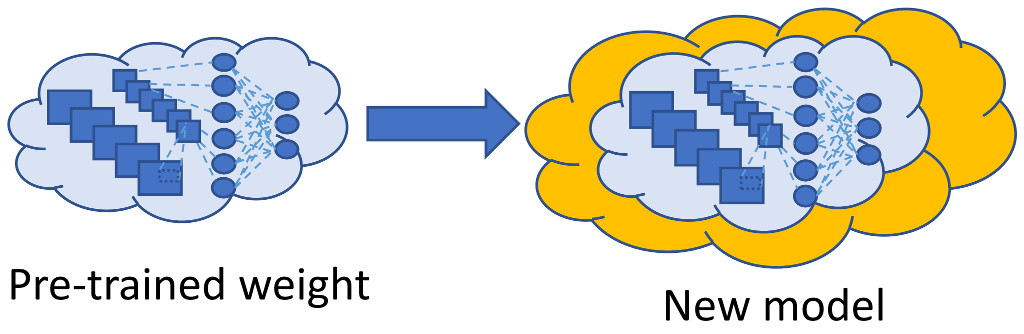

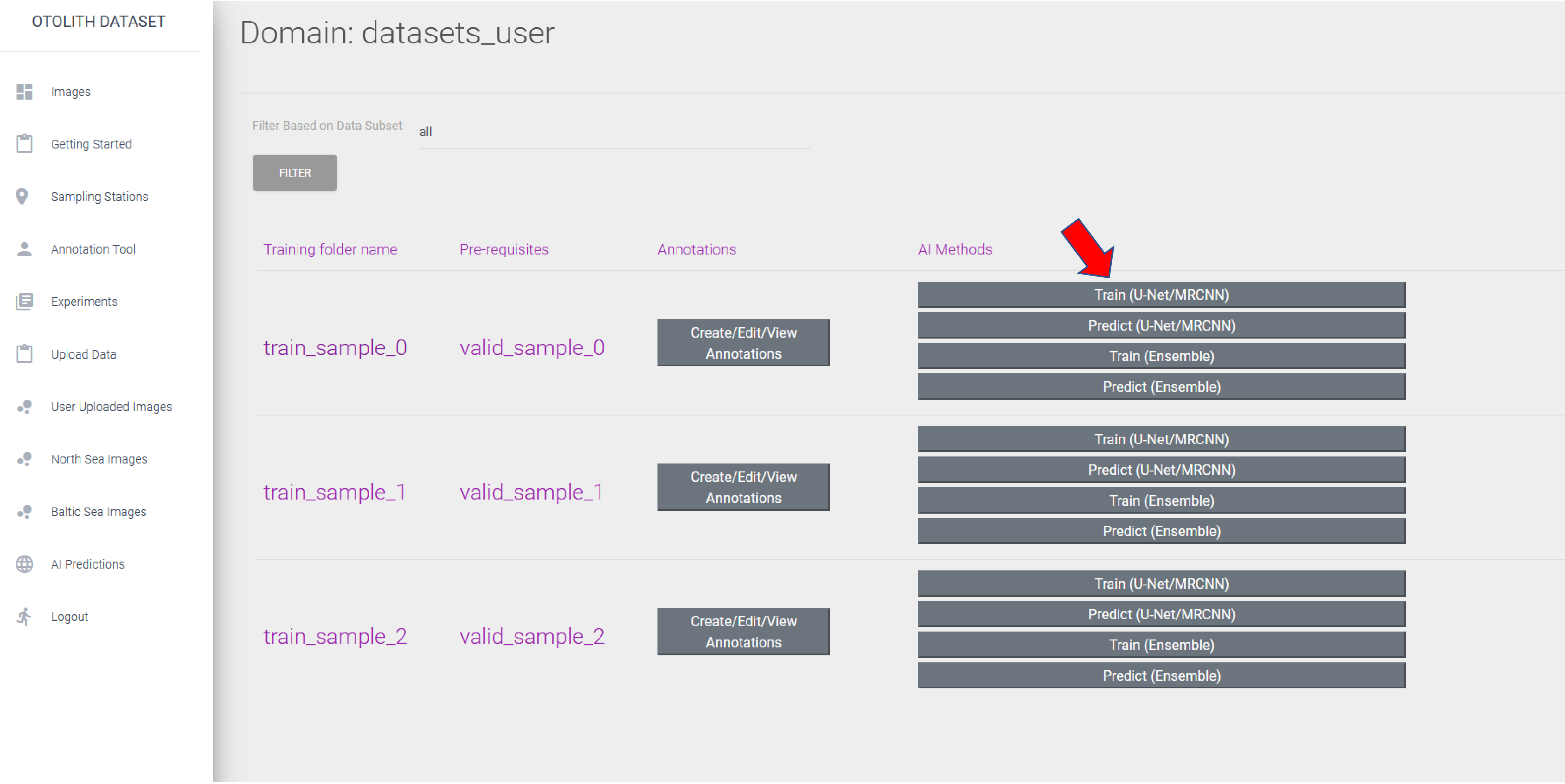
OTOLITH DATASET
Images
Getting Started
Sampling Stations
Annotation Tool
Experiments
Upload Data
User Uploaded Images
North Sea Images
Baltic Sea Images
AI Predictions
Logout
Domain: datasets_user
Filter Based on Data Subset
all
FILTER
Training folder name
Pre-requisites
Annotations
AI Methods
train_sample_0
valid_sample_0
Create/Edit/View Annotations
Train (U-Net/MRCNN)
Predict (U-Net/MRCNN)
Train (Ensemble)
Predict (Ensemble)
train_sample_1
valid_sample_1
Create/Edit/View Annotations
Train (U-Net/MRCNN)
Predict (U-Net/MRCNN)
Train (Ensemble)
Predict (Ensemble)
train_sample_2
valid_sample_2
Create/Edit/View Annotations
Train (U-Net/MRCNN)
Predict (U-Net/MRCNN)
Train (Ensemble)
Predict (Ensemble)

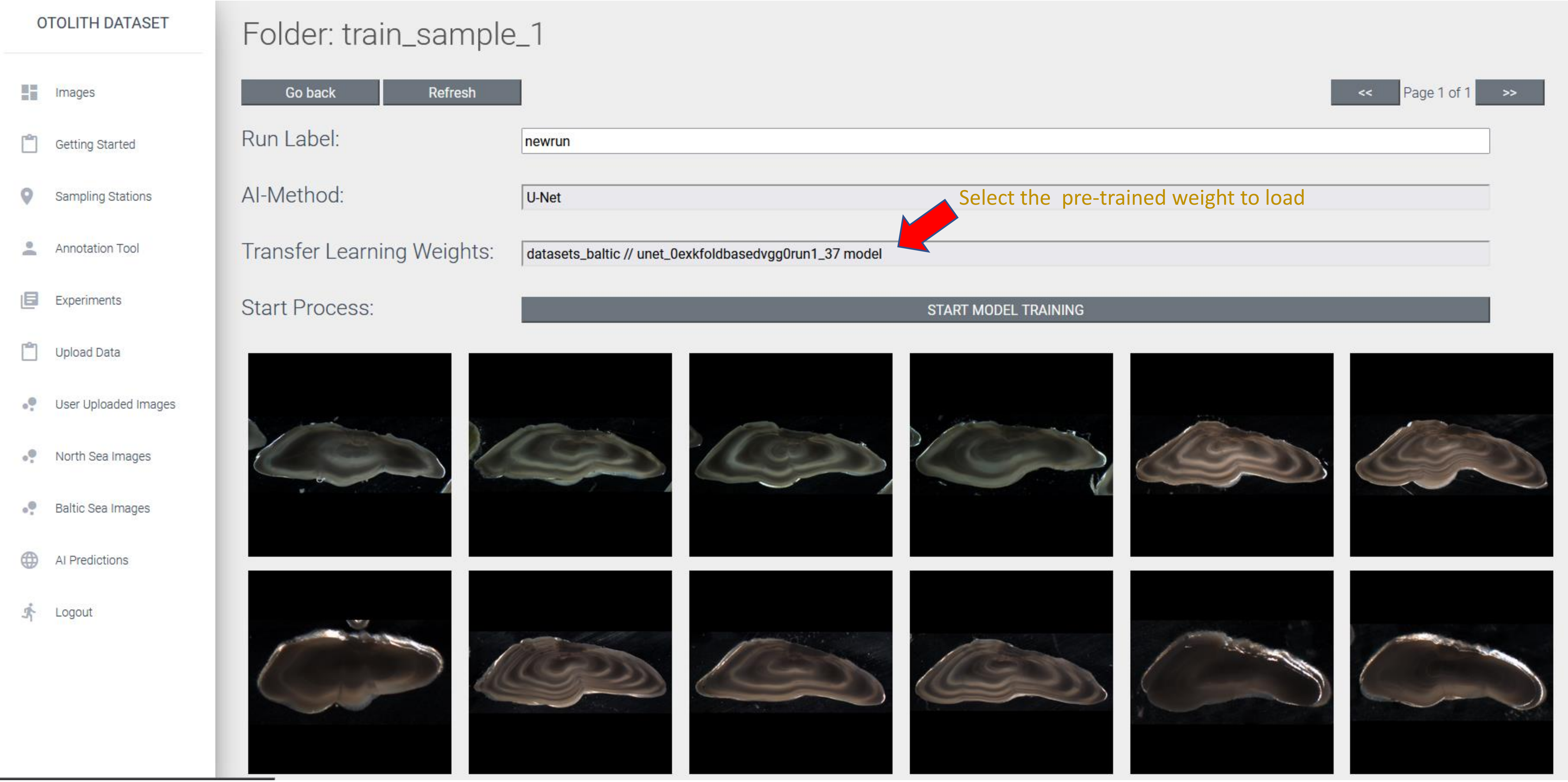
OTOLITH DATASET
Images
Getting Started
Sampling Stations
Annotation Tool
Experiments
Upload Data
User Uploaded Images
North Sea Images
Baltic Sea Images
AI Predictions
Logout
Folder: train_sample_1
Go back
Refresh
<<
Page 1 of 1
>>
Run Label:
newrun
AI-Method:
U-Net
Select the pre-trained weight to load
Transfer Learning Weights:
datasets_baltic // unet_0exkfoldbasedvgg0run1_37 model
Start Process:
START MODEL TRAINING

Ensemble Learning

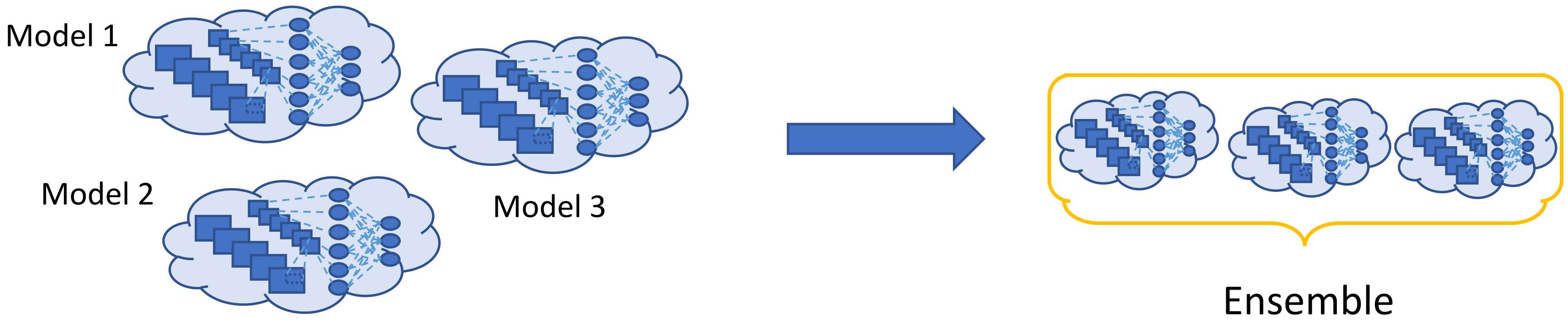

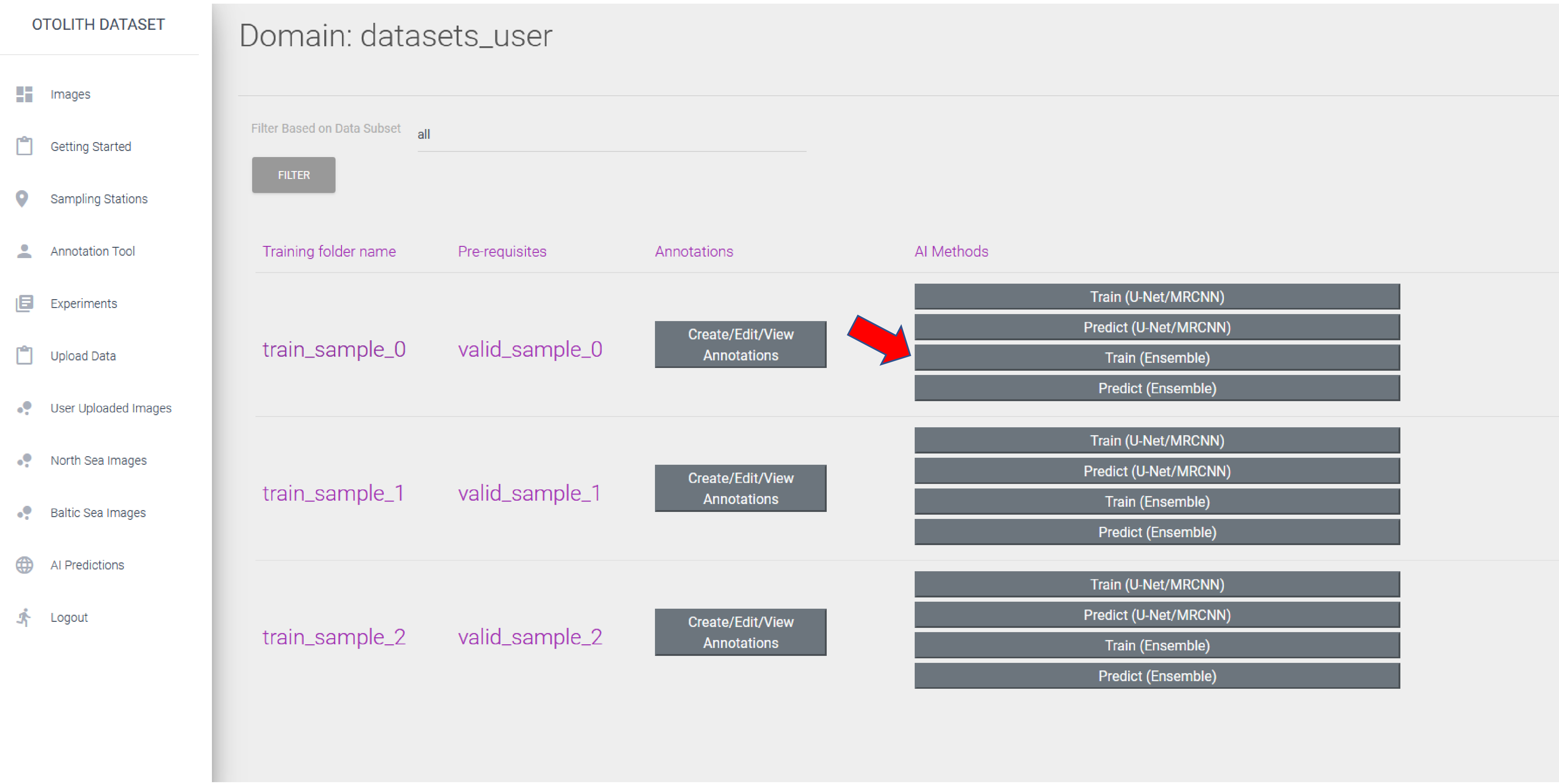
OTOLITH DATASET
Images
Getting Started
Sampling Stations
Annotation Tool
Experiments
Upload Data
User Uploaded Images
North Sea Images
Baltic Sea Images
AI Predictions
Logout
Domain: datasets_user
Filter Based on Data Subset
all
FILTER
Training folder name
Pre-requisites
Annotations
AI Methods
train_sample_0
valid_sample_0
Create/Edit/View Annotations
Train (U-Net/MRCNN)
Predict (U-Net/MRCNN)
Train (Ensemble)
Predict (Ensemble)
train_sample_1
valid_sample_1
Create/Edit/View Annotations
Train (U-Net/MRCNN)
Predict (U-Net/MRCNN)
Train (Ensemble)
Predict (Ensemble)
train_sample_2
valid_sample_2
Create/Edit/View Annotations
Train (U-Net/MRCNN)
Predict (U-Net/MRCNN)
Train (Ensemble)
Predict (Ensemble)

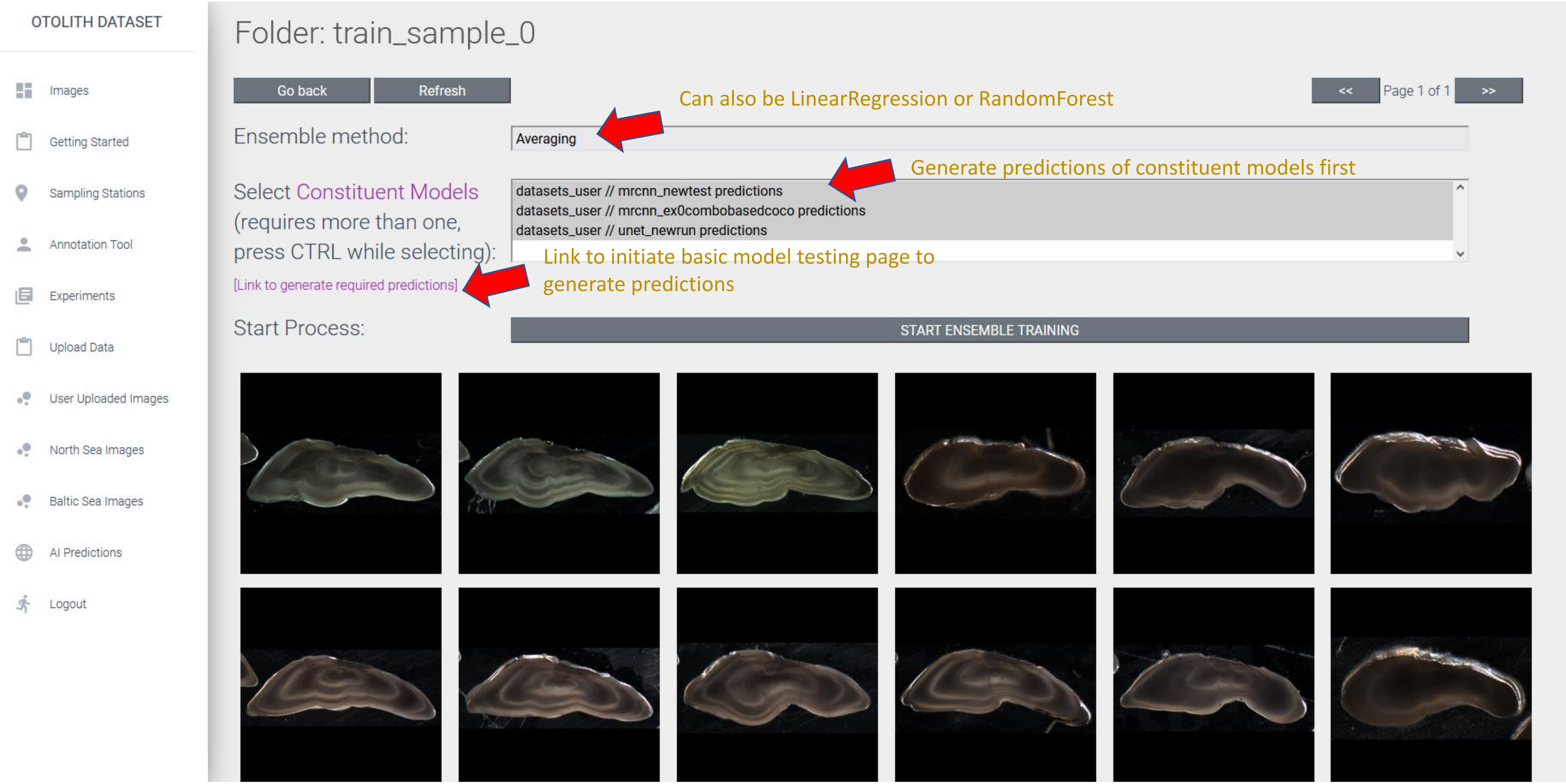
OTOLITH DATASET
Images
Getting Started
Sampling Stations
Annotation Tool
Experiments
Upload Data
User Uploaded Images
North Sea Images
Baltic Sea Images
AI Predictions
Logout
Folder: train_sample_0
Go back
Refresh
<<
Page 1 of 1
>>
Can also be LinearRegression or RandomForest
Ensemble method:
Averaging
Generate predictions of constituent models first
Select Constituent Models (requires more than one, press CTRL while selecting):
datasets_user // mrcnn_newtest predictions
datasets_user // mrcnn_ex0combobasedcoco predictions
datasets_user // unet_newrun predictions
[Link to generate required predictions]
Link to initiate basic model testing page to generate predictions
Start Process:
START ENSEMBLE TRAINING

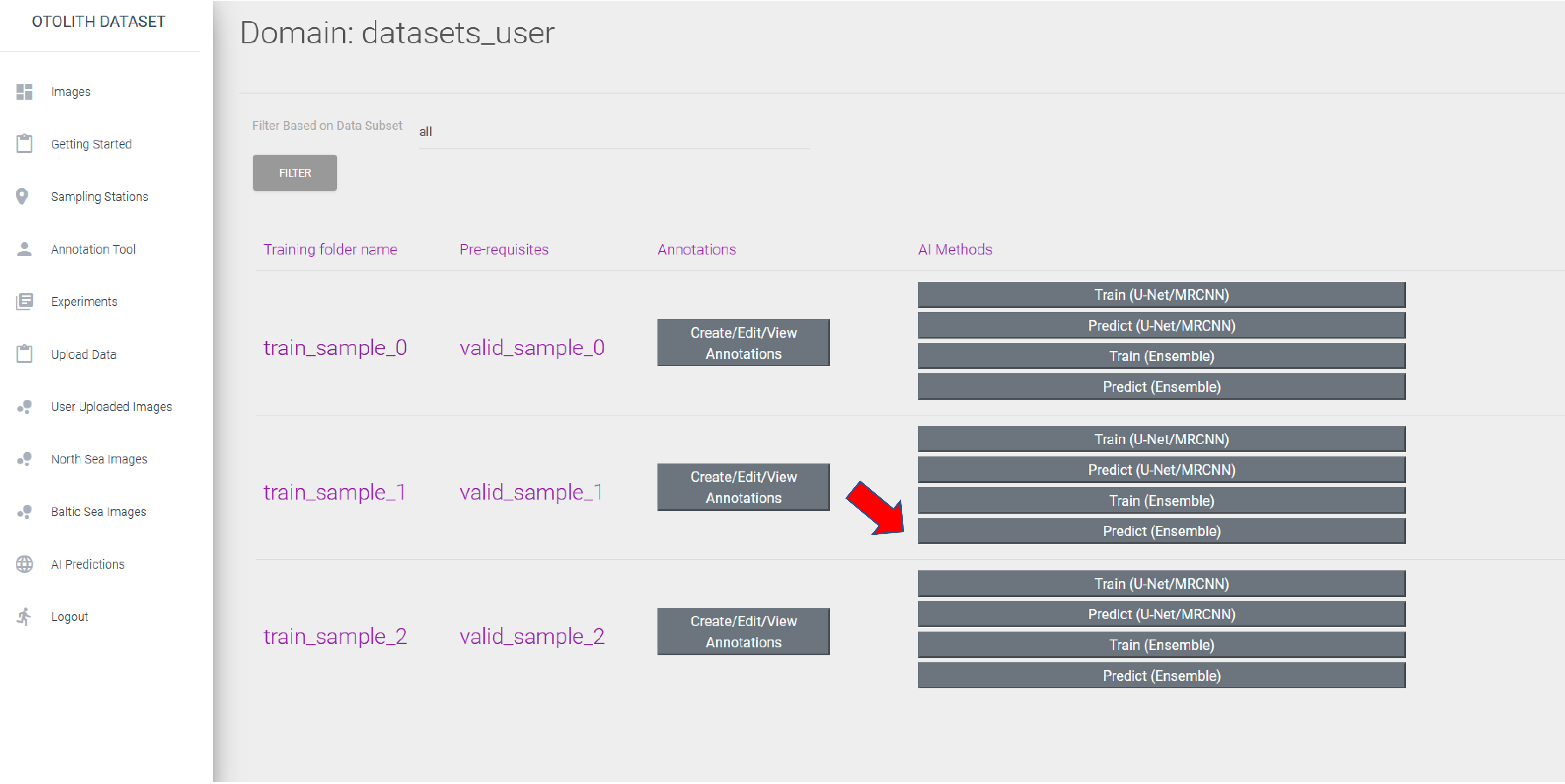
OTOLITH DATASET
Images
Getting Started
Sampling Stations
Annotation Tool
Experiments
Upload Data
User Uploaded Images
North Sea Images
Baltic Sea Images
AI Predictions
Logout
Domain: datasets_user
Filter Based on Data Subset
all
FILTER
Training folder name
Pre-requisites
Annotations
AI Methods
train_sample_0
valid_sample_0
Create/Edit/View Annotations
Train (U-Net/MRCNN)
Predict (U-Net/MRCNN)
Train (Ensemble)
Predict (Ensemble)
train_sample_1
valid_sample_1
Create/Edit/View Annotations
Train (U-Net/MRCNN)
Predict (U-Net/MRCNN)
Train (Ensemble)
Predict (Ensemble)
train_sample_2
valid_sample_2
Create/Edit/View Annotations
Train (U-Net/MRCNN)
Predict (U-Net/MRCNN)
Train (Ensemble)
Predict (Ensemble)

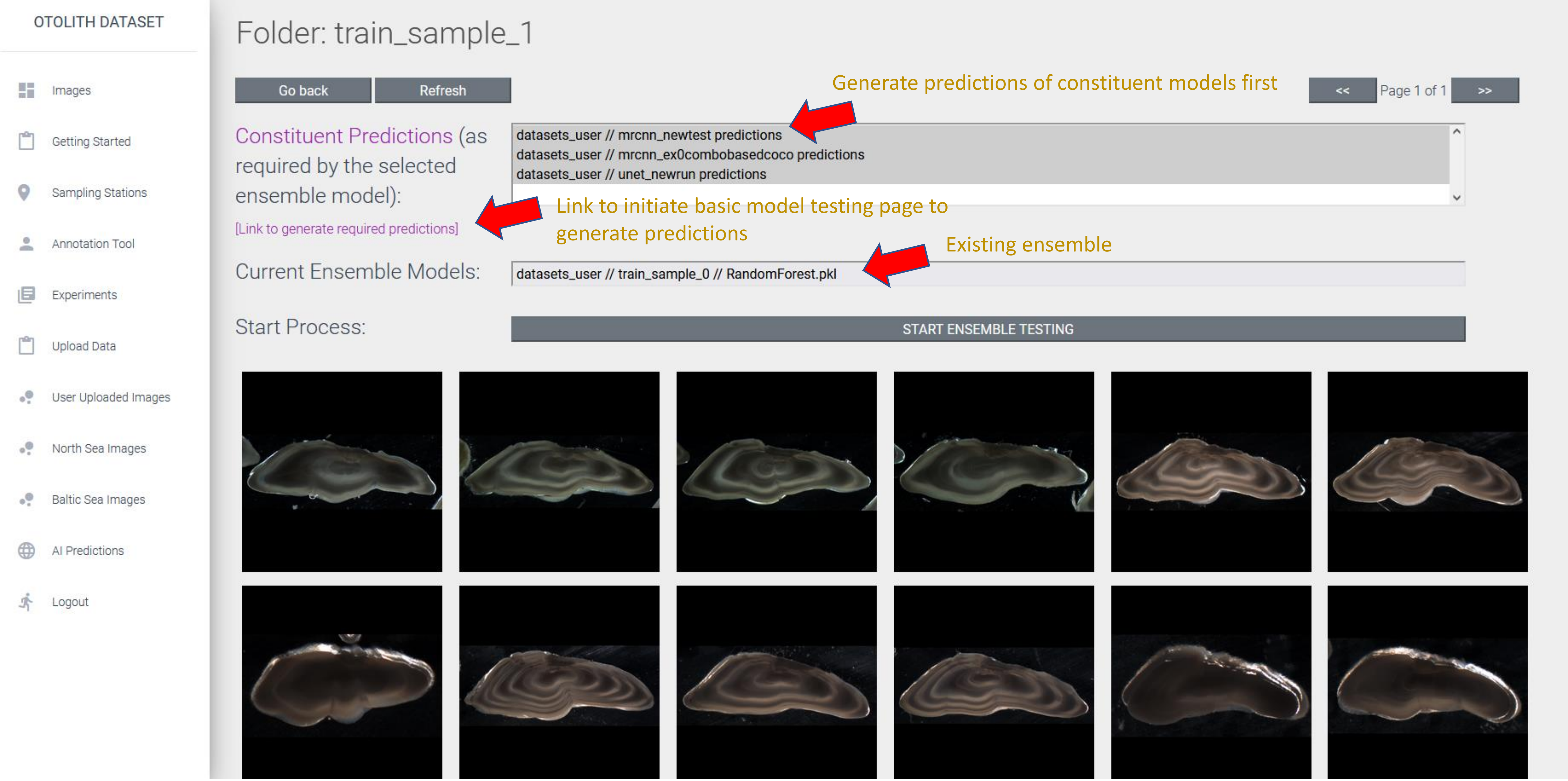
OTOLITH DATASET
Images
Getting Started
Sampling Stations
Annotation Tool
Experiments
Upload Data
User Uploaded Images
North Sea Images
Baltic Sea Images
AI Predictions
Logout
Folder: train_sample_1
Go back
Refresh
Generate predictions of constituent models first
<<
Page 1 of 1
>>
Constituent Predictions (as required by the selected ensemble model):
datasets_user // mrcnn_newtest predictions
datasets_user // mrcnn_ex0combobasedcoco predictions
datasets_user // unet_newrun predictions
[Link to generate required predictions]
Link to initiate basic model testing page to generate predictions
Existing ensemble
Current Ensemble Models:
datasets_user // train_sample_0 // RandomForest.pkl
Start Process:
START ENSEMBLE TESTING

OTOLITH DATASET

- Images
- Getting Started
- Sampling Stations
- Annotation Tool
- Experiments
- Upload Data
- User Uploaded Images
- North Sea Images
- Baltic Sea Images
- AI Predictions
- Logout

Section: Datasets

Image Name	AI Reading (87%)	Manual Reading
16_716_12.png	2	2
16_716_45.png	3	3
16_716_46.png	3	3
16_716_66.png	2	2
17_731_108.png	4	4
17_731_116.png	5	5
17_731_152.png	1	1
17_731_181.png	5	5
17_731_192.png	3	3
17_731_213.png	4	4
17_731_248.png	1	1
17_731_249.png	1	1
17_731_304.png	1	1
17_731_385.png	1	1
17_731_86.png	1	1

Continual Learning

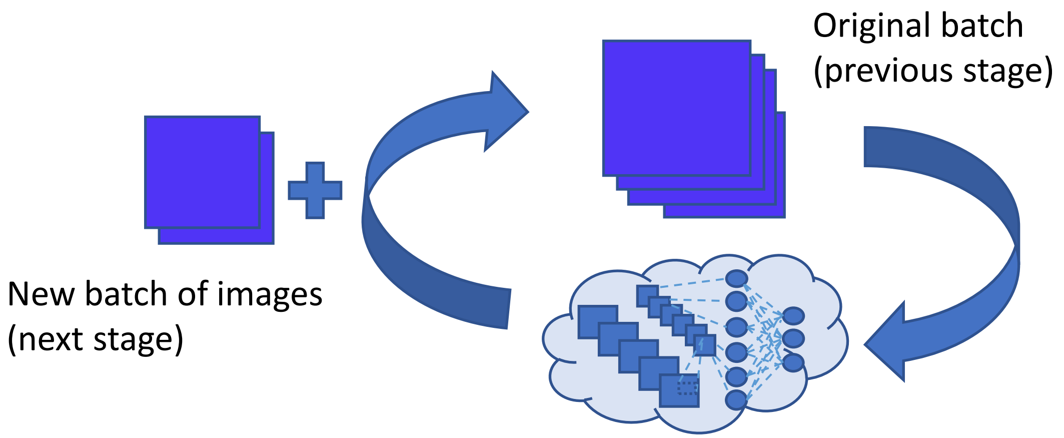

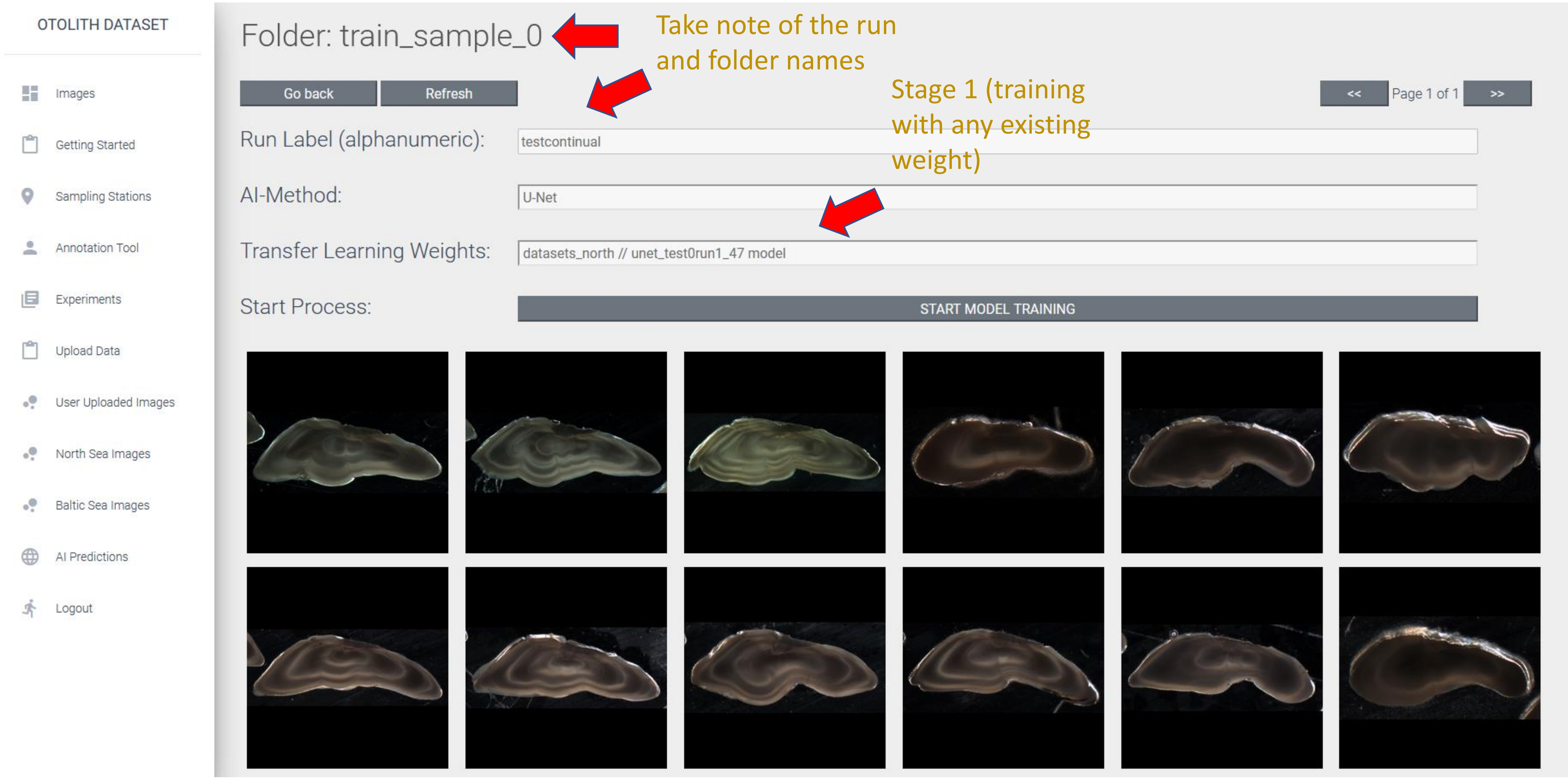
OTOLITH DATASET
Images
Getting Started
Sampling Stations
Annotation Tool
Experiments
Upload Data
User Uploaded Images
North Sea Images
Baltic Sea Images
AI Predictions
Logout
Folder: train_sample_0
Take note of the run and folder names
Go back
Refresh
Stage 1 (training with any existing weight)
<<
Page 1 of 1
>>
Run Label (alphanumeric):
testcontinual
AI-Method:
U-Net
Transfer Learning Weights:
datasets_north // unet_test0run1_47 model
Start Process:
START MODEL TRAINING

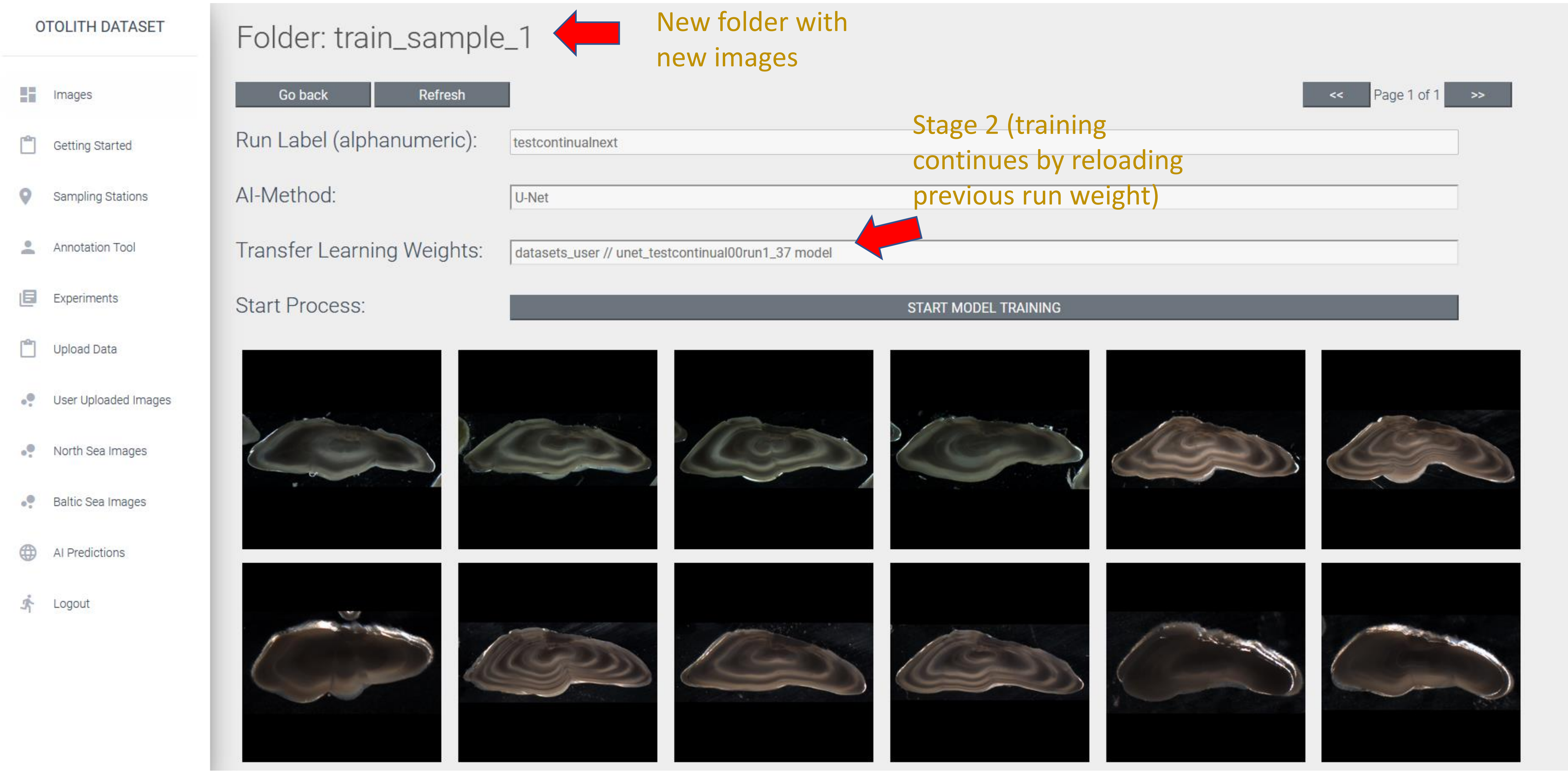
OTOLITH DATASET
Images
Getting Started
Sampling Stations
Annotation Tool
Experiments
Upload Data
User Uploaded Images
North Sea Images
Baltic Sea Images
AI Predictions
Logout
Folder: train_sample_1
New folder with new images
Go back
Refresh
<<
Page 1 of 1
>>
Run Label (alphanumeric):
testcontinualnext
Stage 2 (training continues by reloading previous run weight)
AI-Method:
U-Net
Transfer Learning Weights:
datasets_user // unet_testcontinual00run1_37 model
Start Process:
START MODEL TRAINING

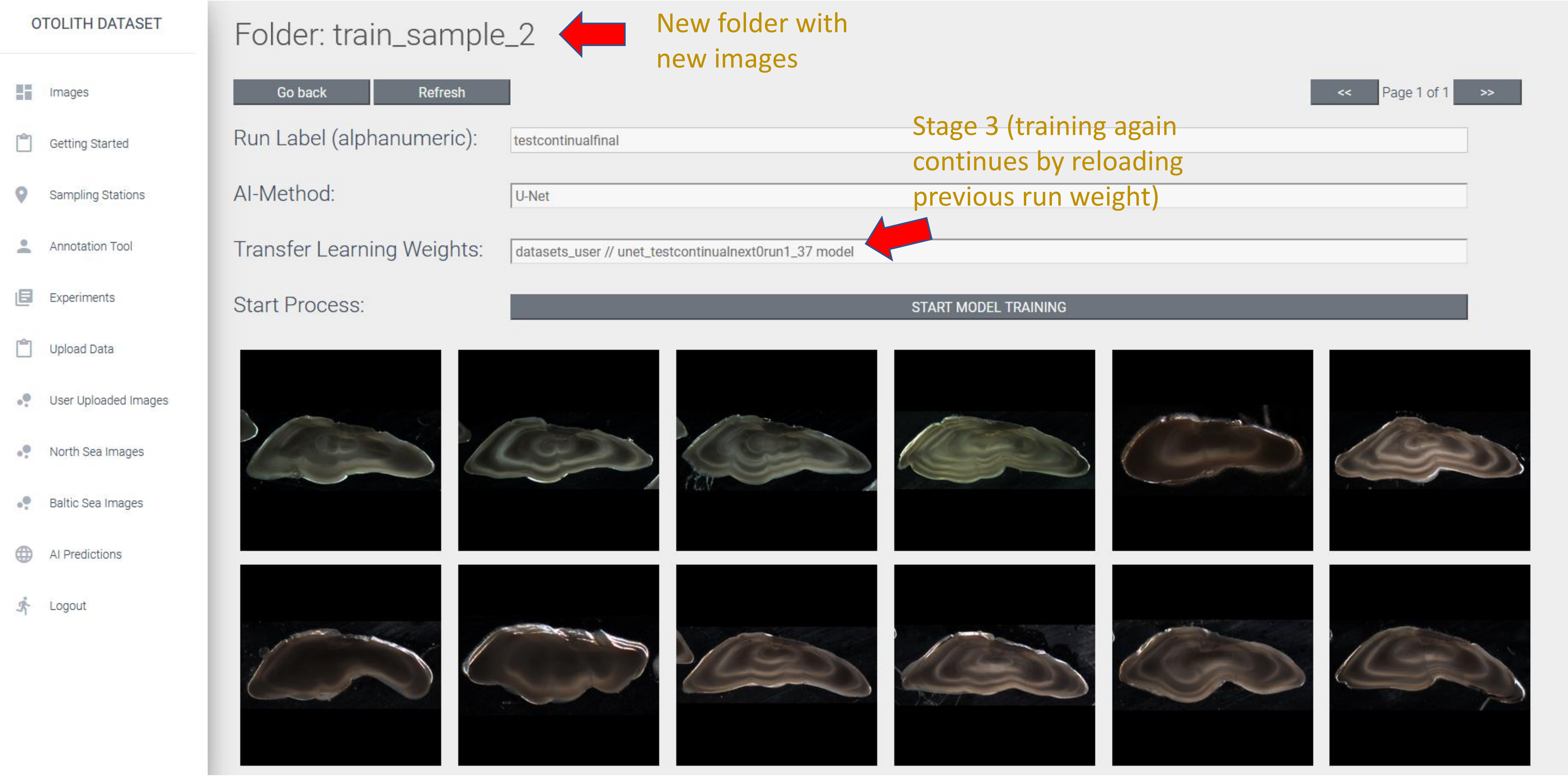
OTOLITH DATASET
Images
Getting Started
Sampling Stations
Annotation Tool
Experiments
Upload Data
User Uploaded Images
North Sea Images
Baltic Sea Images
AI Predictions
Logout
Folder: train_sample_2
New folder with new images
Go back
Refresh
<<
Page 1 of 1
>>
Run Label (alphanumeric):
testcontinualfinal
Stage 3 (training again continues by reloading previous run weight)
AI-Method:
U-Net
Transfer Learning Weights:
datasets_user // unet_testcontinualnext0run1_37 model
Start Process:
START MODEL TRAINING
