# Supplementary material for: An interactive AI-driven platform for fish age reading
Source: PLoS One. 2024 Nov 18;19(11):e0313934. doi: 10.1371/journal.pone.0313934 (PMC11573220; doi:10.1371/journal.pone.0313934)
Supplement: S1 File — (ZIP) [file pone.0313934.s003.zip › USER_MANUAL/UserManual_BasicAI.pdf]

Basic Training

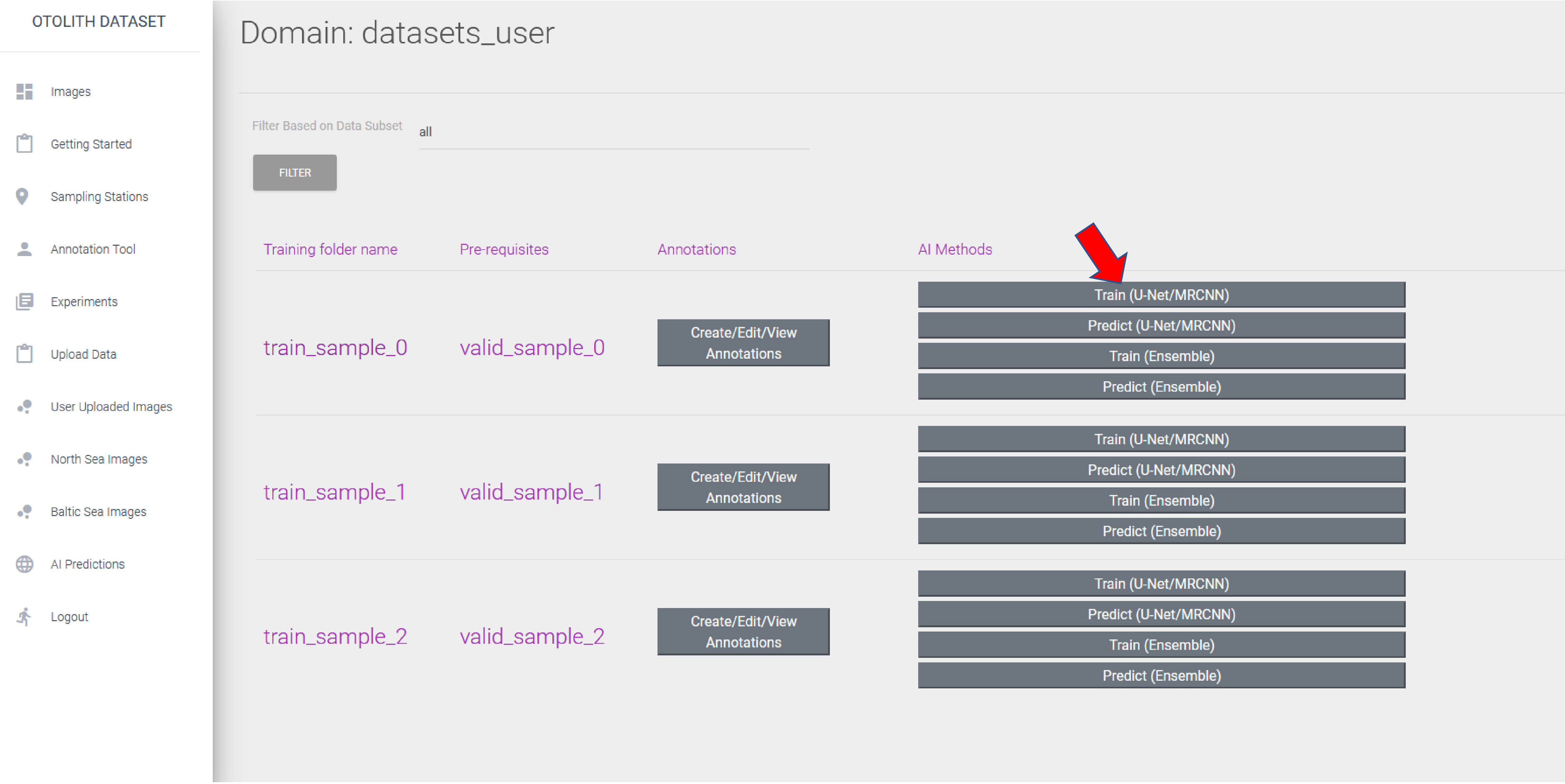
OTOLITH DATASET
Images
Getting Started
Sampling Stations
Annotation Tool
Experiments
Upload Data
User Uploaded Images
North Sea Images
Baltic Sea Images
AI Predictions
Logout
Domain: datasets_user
Filter Based on Data Subset
all
FILTER
Training folder name
Pre-requisites
Annotations
AI Methods
train_sample_0
valid_sample_0
Create/Edit/View Annotations
Train (U-Net/MRCNN)
Predict (U-Net/MRCNN)
Train (Ensemble)
Predict (Ensemble)
train_sample_1
valid_sample_1
Create/Edit/View Annotations
Train (U-Net/MRCNN)
Predict (U-Net/MRCNN)
Train (Ensemble)
Predict (Ensemble)
train_sample_2
valid_sample_2
Create/Edit/View Annotations
Train (U-Net/MRCNN)
Predict (U-Net/MRCNN)
Train (Ensemble)
Predict (Ensemble)

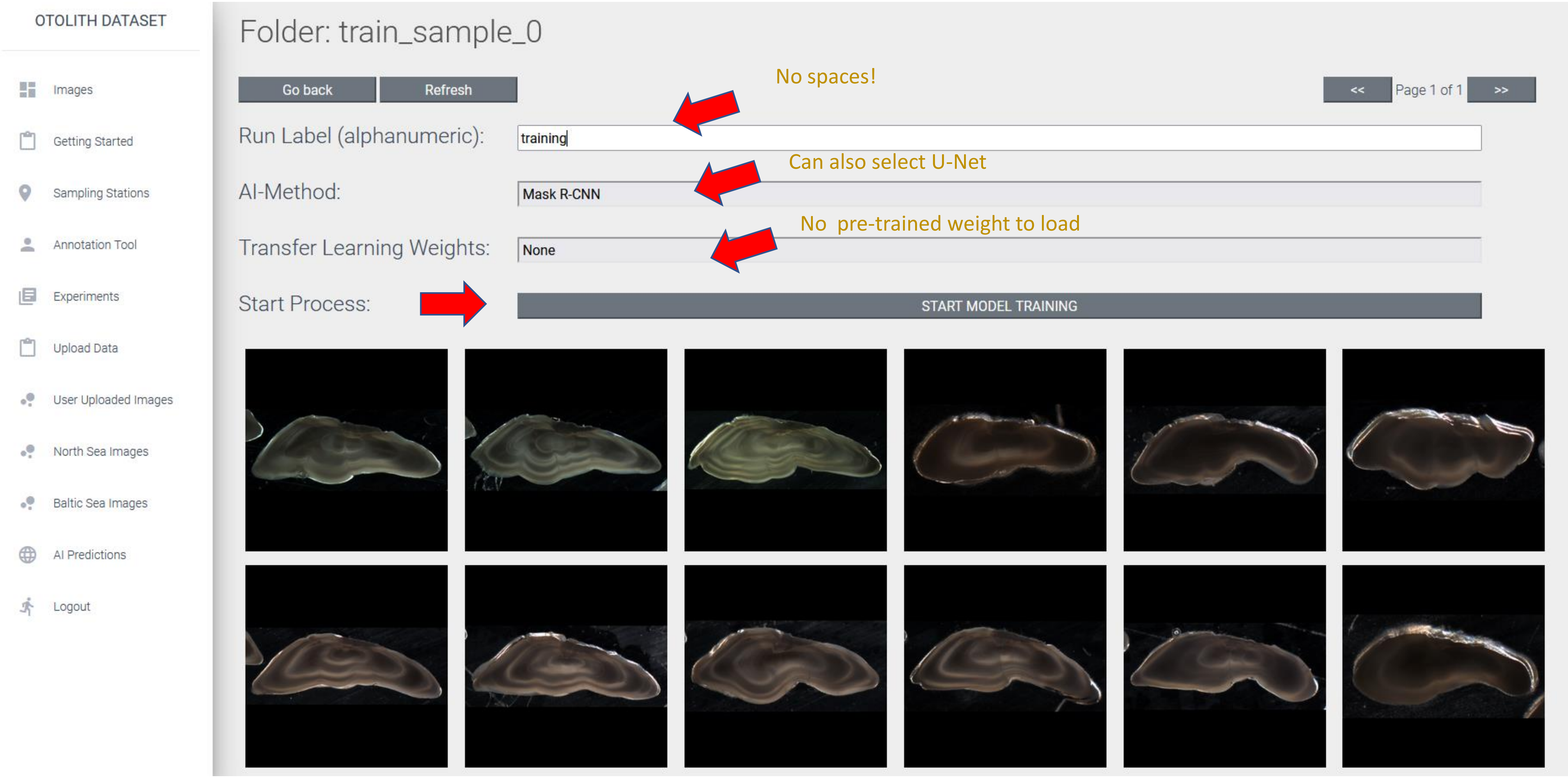
OTOLITH DATASET
Images
Getting Started
Sampling Stations
Annotation Tool
Experiments
Upload Data
User Uploaded Images
North Sea Images
Baltic Sea Images
AI Predictions
Logout
Folder: train_sample_0
Go back
Refresh
No spaces!
<<
Page 1 of 1
>>
Run Label (alphanumeric):
training
Can also select U-Net
AI-Method:
Mask R-CNN
No pre-trained weight to load
Transfer Learning Weights:
None
Start Process:
START MODEL TRAINING

Basic Testing

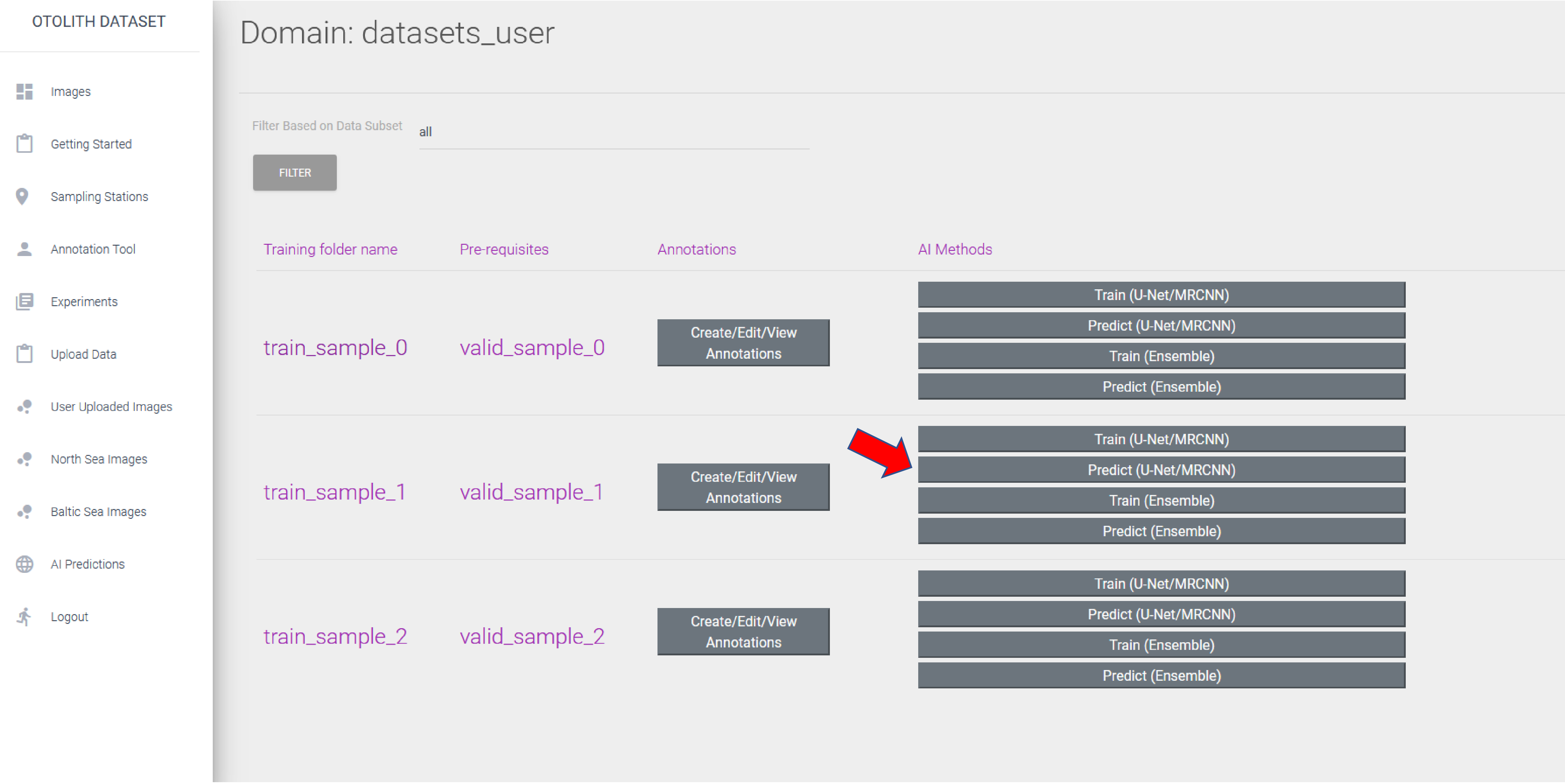
OTOLITH DATASET
Images
Getting Started
Sampling Stations
Annotation Tool
Experiments
Upload Data
User Uploaded Images
North Sea Images
Baltic Sea Images
AI Predictions
Logout
Domain: datasets_user
Filter Based on Data Subset
all
FILTER
Training folder name
Pre-requisites
Annotations
AI Methods
train_sample_0
valid_sample_0
Create/Edit/View Annotations
Train (U-Net/MRCNN)
Predict (U-Net/MRCNN)
Train (Ensemble)
Predict (Ensemble)
train_sample_1
valid_sample_1
Create/Edit/View Annotations
Train (U-Net/MRCNN)
Predict (U-Net/MRCNN)
Train (Ensemble)
Predict (Ensemble)
train_sample_2
valid_sample_2
Create/Edit/View Annotations
Train (U-Net/MRCNN)
Predict (U-Net/MRCNN)
Train (Ensemble)
Predict (Ensemble)

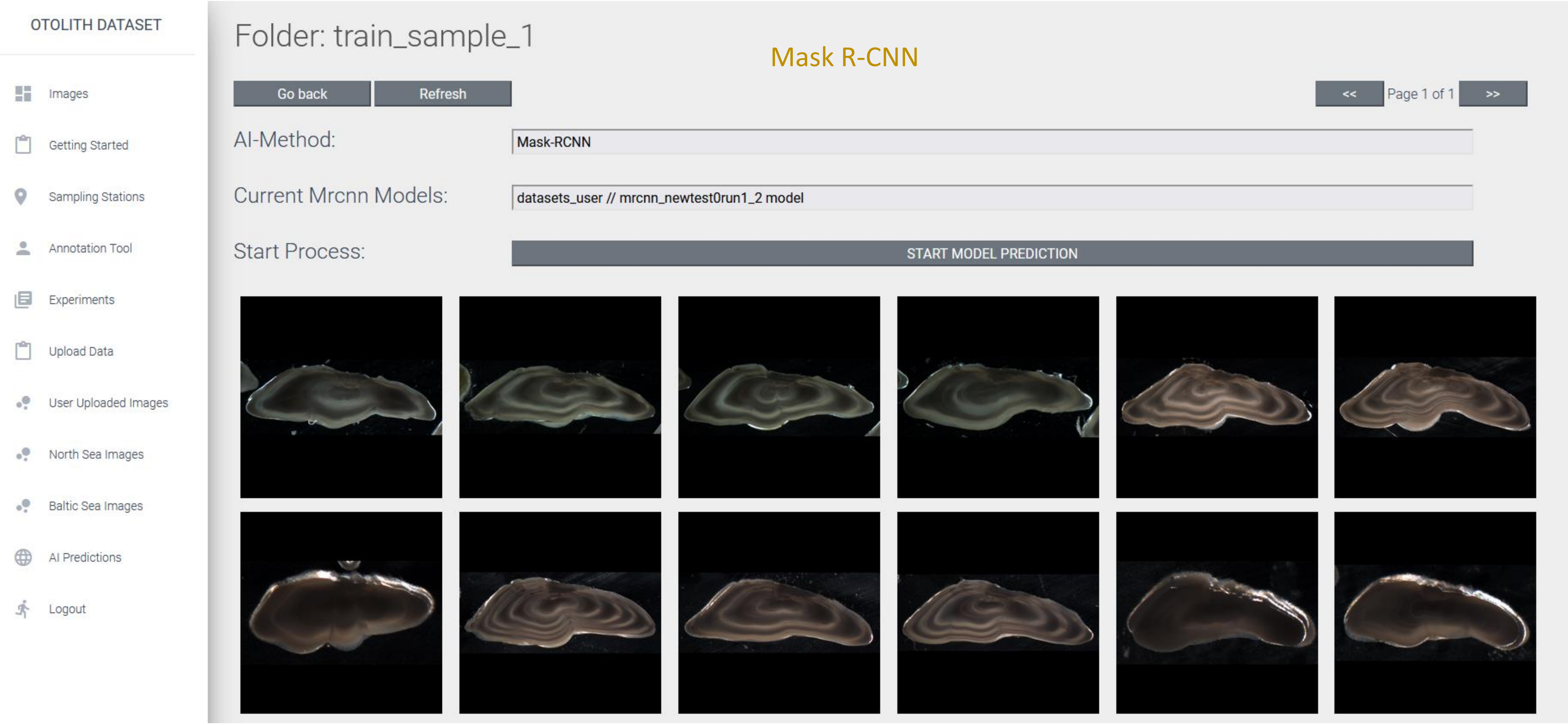
OTOLITH DATASET
Images
Getting Started
Sampling Stations
Annotation Tool
Experiments
Upload Data
User Uploaded Images
North Sea Images
Baltic Sea Images
AI Predictions
Logout
Folder: train_sample_1
Mask R-CNN
Go back
Refresh
<<
Page 1 of 1
>>
AI-Method:
Mask-RCNN
Current Mrcnn Models:
datasets_user // mrcnn_newtest0run1_2 model
Start Process:
START MODEL PREDICTION

Mask R-CNN

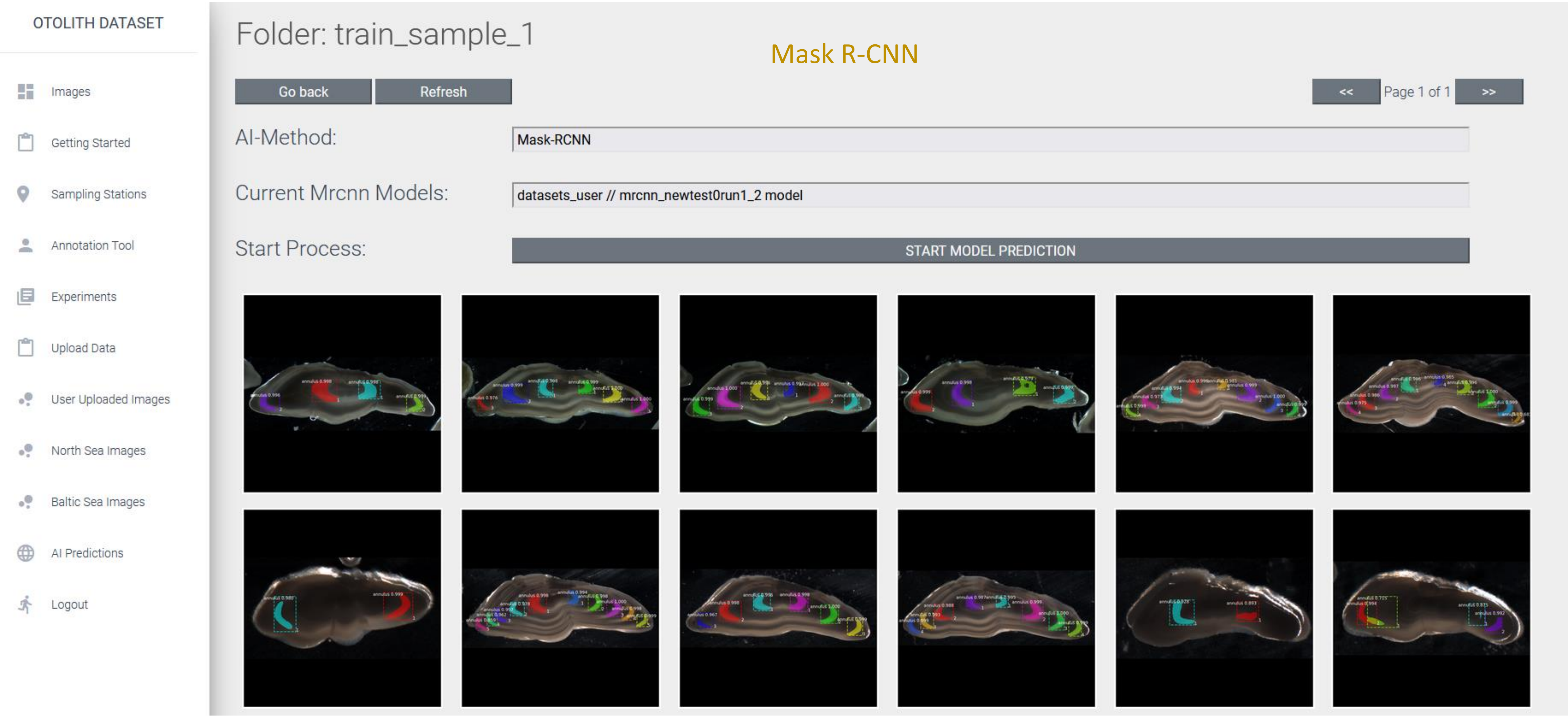
OTOLITH DATASET
Images
Getting Started
Sampling Stations
Annotation Tool
Experiments
Upload Data
User Uploaded Images
North Sea Images
Baltic Sea Images
AI Predictions
Logout
Folder: train_sample_1
Go back
Refresh
<<
Page 1 of 1
>>
AI-Method:
Mask-RCNN
Current Mrcnn Models:
datasets_user // mrcnn_newtest0run1_2 model
Start Process:
START MODEL PREDICTION

U-Net

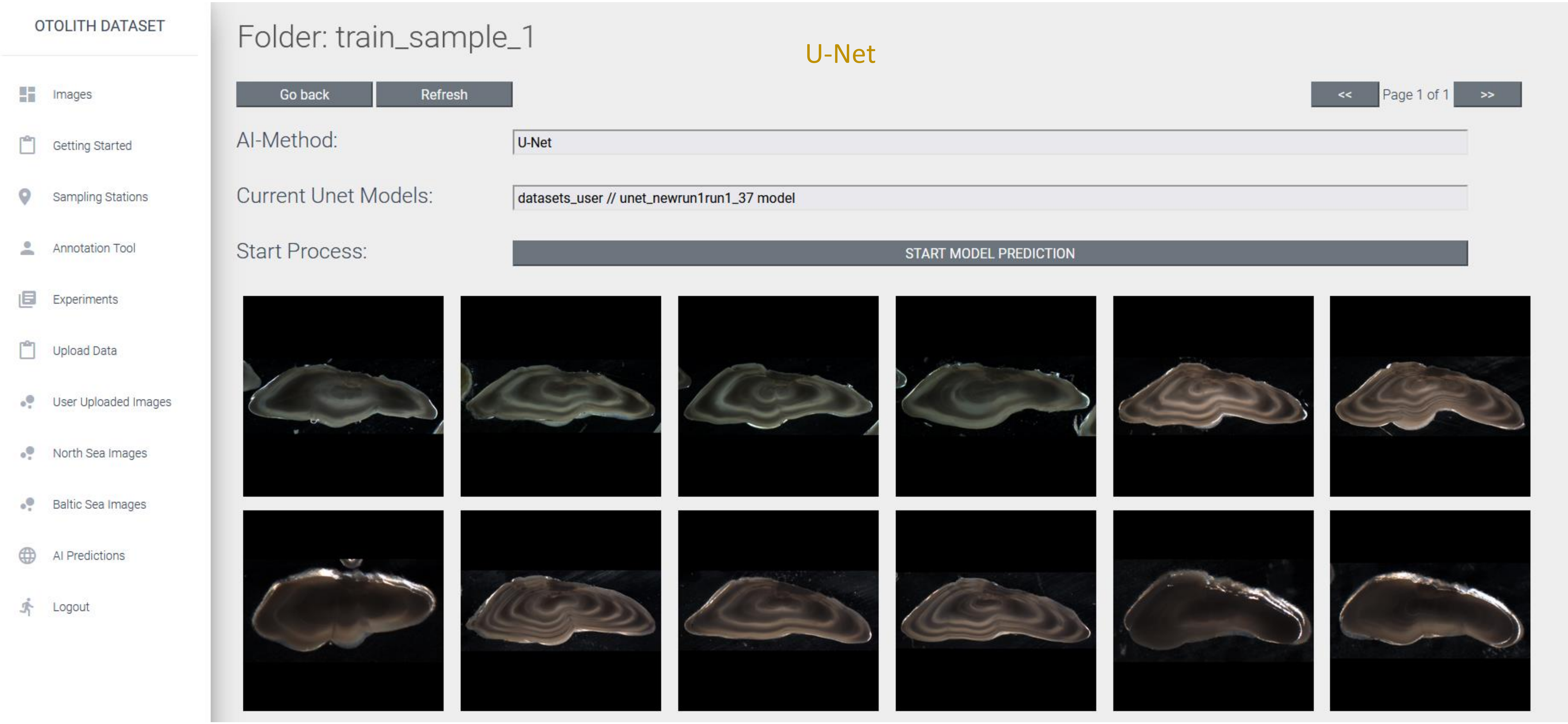
OTOLITH DATASET
Images
Getting Started
Sampling Stations
Annotation Tool
Experiments
Upload Data
User Uploaded Images
North Sea Images
Baltic Sea Images
AI Predictions
Logout
Folder: train_sample_1
Go back
Refresh
<<
Page 1 of 1
>>
AI-Method:
U-Net
Current Unet Models:
datasets_user // unet_newrun1run1_37 model
Start Process:
START MODEL PREDICTION

U-Net

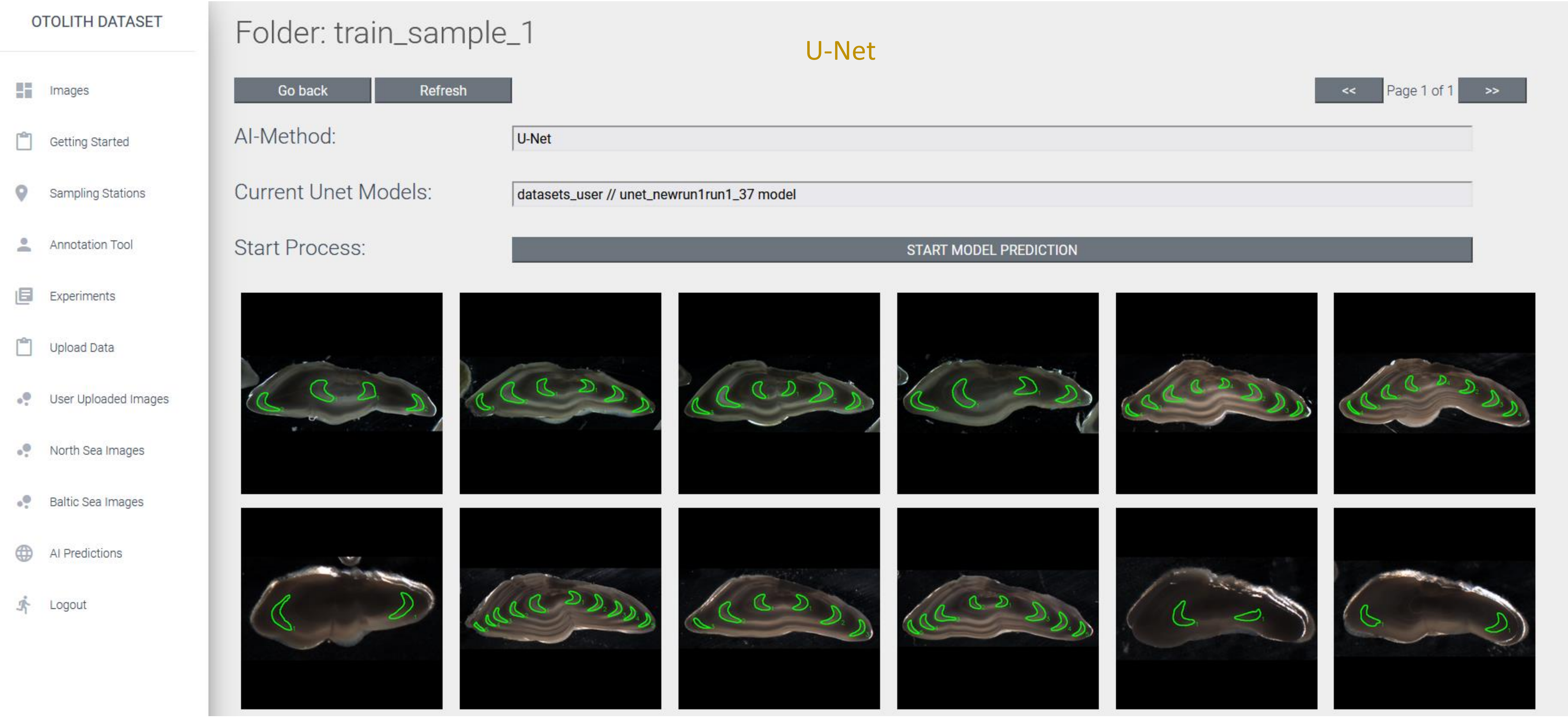
OTOLITH DATASET
Images
Getting Started
Sampling Stations
Annotation Tool
Experiments
Upload Data
User Uploaded Images
North Sea Images
Baltic Sea Images
AI Predictions
Logout
Folder: train_sample_1
Go back
Refresh
<<
Page 1 of 1
>>
AI-Method:
U-Net
Current Unet Models:
datasets_user // unet_newrun1run1_37 model
Start Process:
START MODEL PREDICTION
