# Supplementary material for: An interactive AI-driven platform for fish age reading
Source: PLoS One. 2024 Nov 18;19(11):e0313934. doi: 10.1371/journal.pone.0313934 (PMC11573220; doi:10.1371/journal.pone.0313934)

Uploading of image files and age metadata

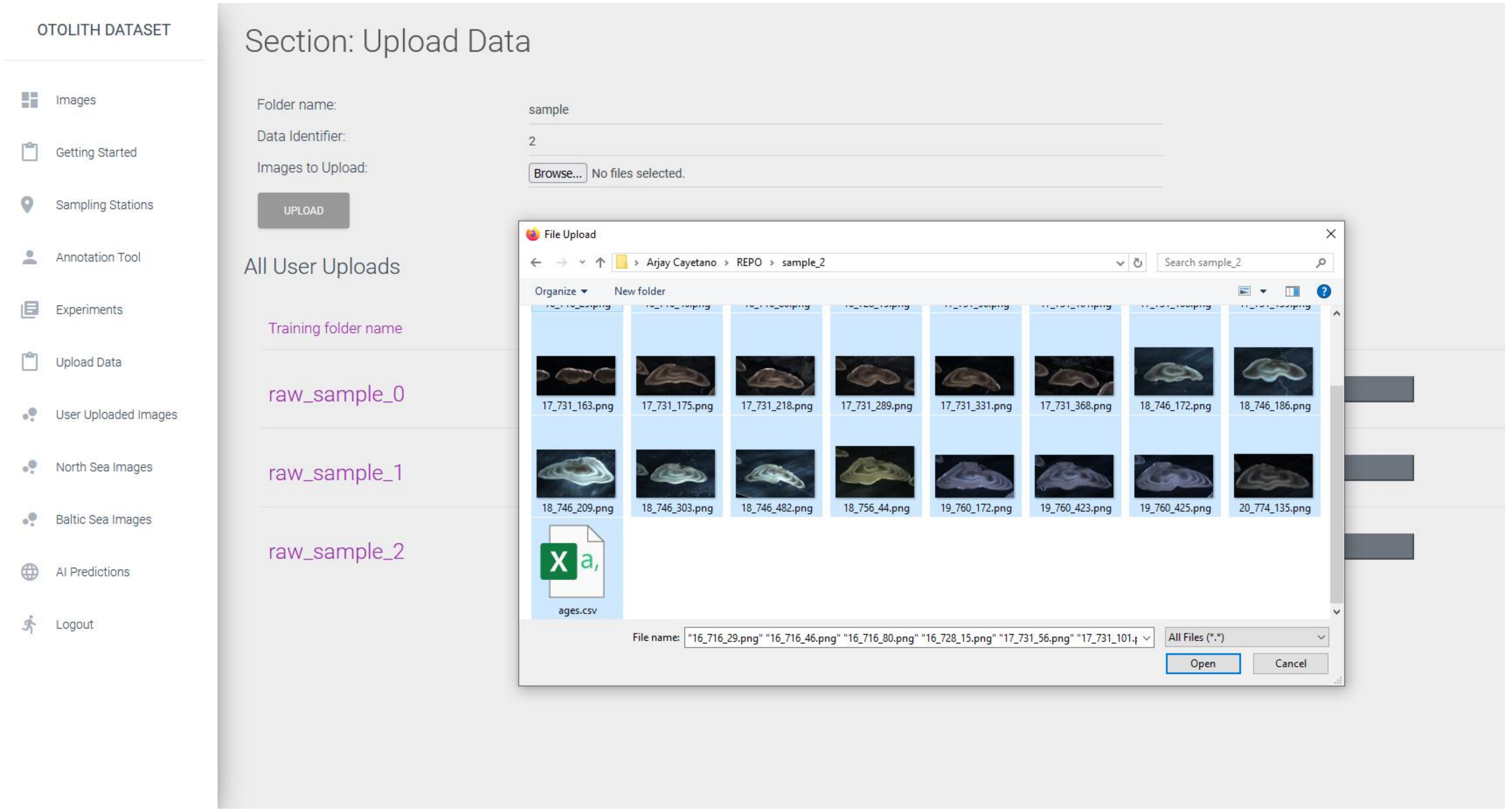
OTOLITH DATASET
Images
Getting Started
Sampling Stations
Annotation Tool
Experiments
Upload Data
User Uploaded Images
North Sea Images
Baltic Sea Images
AI Predictions
Logout
Section: Upload Data
Folder name:
sample
Data Identifier:
2
Images to Upload:
Browse... No files selected.
UPLOAD
All User Uploads
Training folder name
raw_sample_0
raw_sample_1
raw_sample_2
File Upload
Arjay Cayetano › REPO › sample_2
Search sample_2
Organize
New folder
17_731_163.png
17_731_175.png
17_731_218.png
17_731_289.png
17_731_331.png
17_731_368.png
18_746_172.png
18_746_186.png
18_746_209.png
18_746_303.png
18_746_482.png
18_756_44.png
19_760_172.png
19_760_423.png
19_760_425.png
20_774_135.png
ages.csv
File name:
"16_716_29.png" "16_716_46.png" "16_716_80.png" "16_728_15.png" "17_731_56.png" "17_731_101.
All Files (*.*)
Open
Cancel

Notes

- Images (png format) to be uploaded should be selected (CTRL+click)
- Comma-separated age metadata file (e.g. ages.csv) should be included
  - No header and with the following format: ‘name-of-file,age’
    - 17_101_10.png,5
    - 17_101_10.png,5
- It will uploaded with the corresponding raw folder
  - E.g. raw_sample_2

Processing raw folder to generate training folder

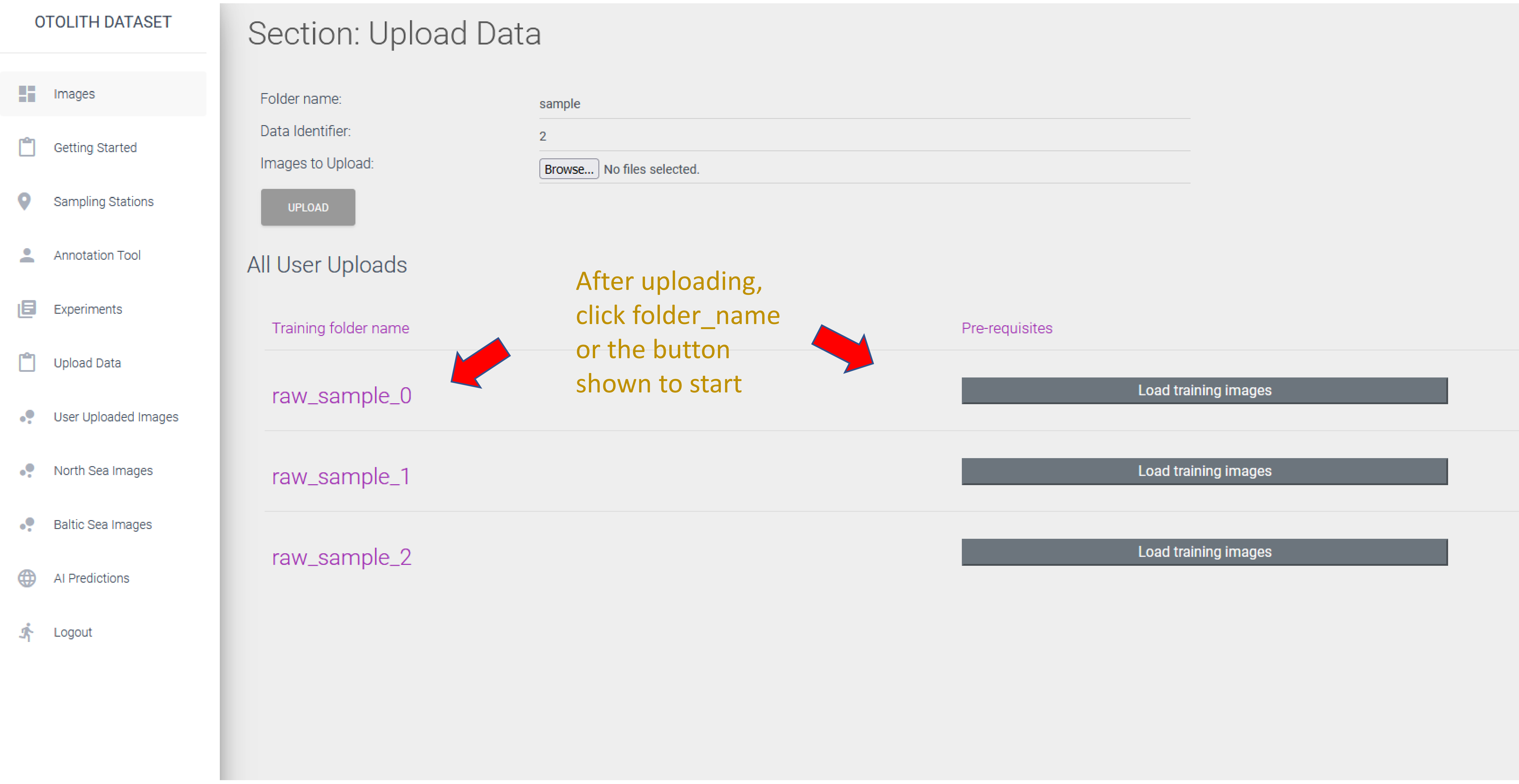
OTOLITH DATASET
Images
Getting Started
Sampling Stations
Annotation Tool
Experiments
Upload Data
User Uploaded Images
North Sea Images
Baltic Sea Images
AI Predictions
Logout
Section: Upload Data
Folder name:
sample
Data Identifier:
2
Images to Upload:
Browse... No files selected.
UPLOAD
All User Uploads
After uploading, click folder_name or the button shown to start
Training folder name
Pre-requisites
raw_sample_0
Load training images
raw_sample_1
Load training images
raw_sample_2
Load training images

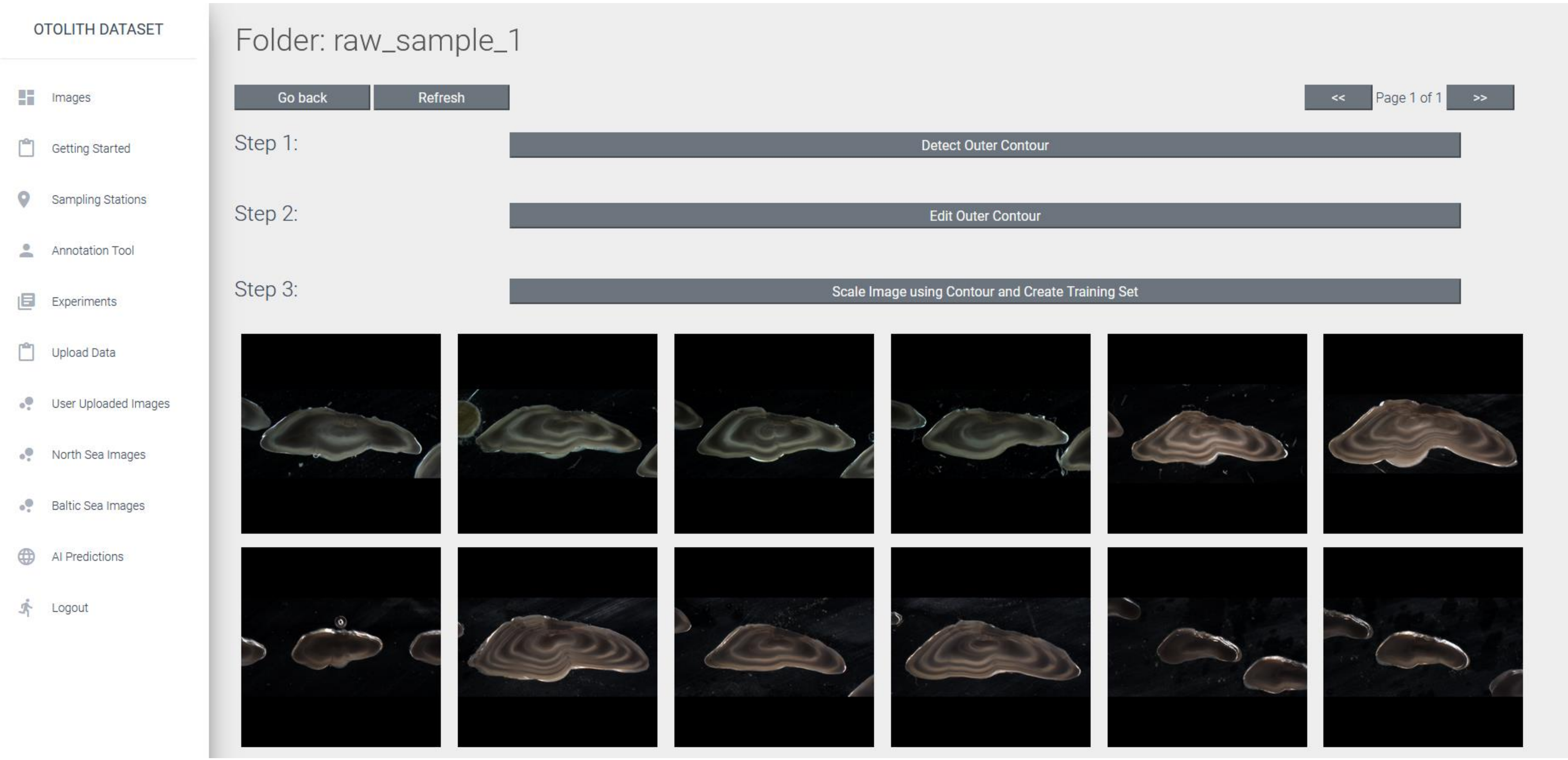
OTOLITH DATASET
Images
Getting Started
Sampling Stations
Annotation Tool
Experiments
Upload Data
User Uploaded Images
North Sea Images
Baltic Sea Images
AI Predictions
Logout
Folder: raw_sample_1
Go back
Refresh
<<
Page 1 of 1
>>
Step 1:
Detect Outer Contour
Step 2:
Edit Outer Contour
Step 3:
Scale Image using Contour and Create Training Set

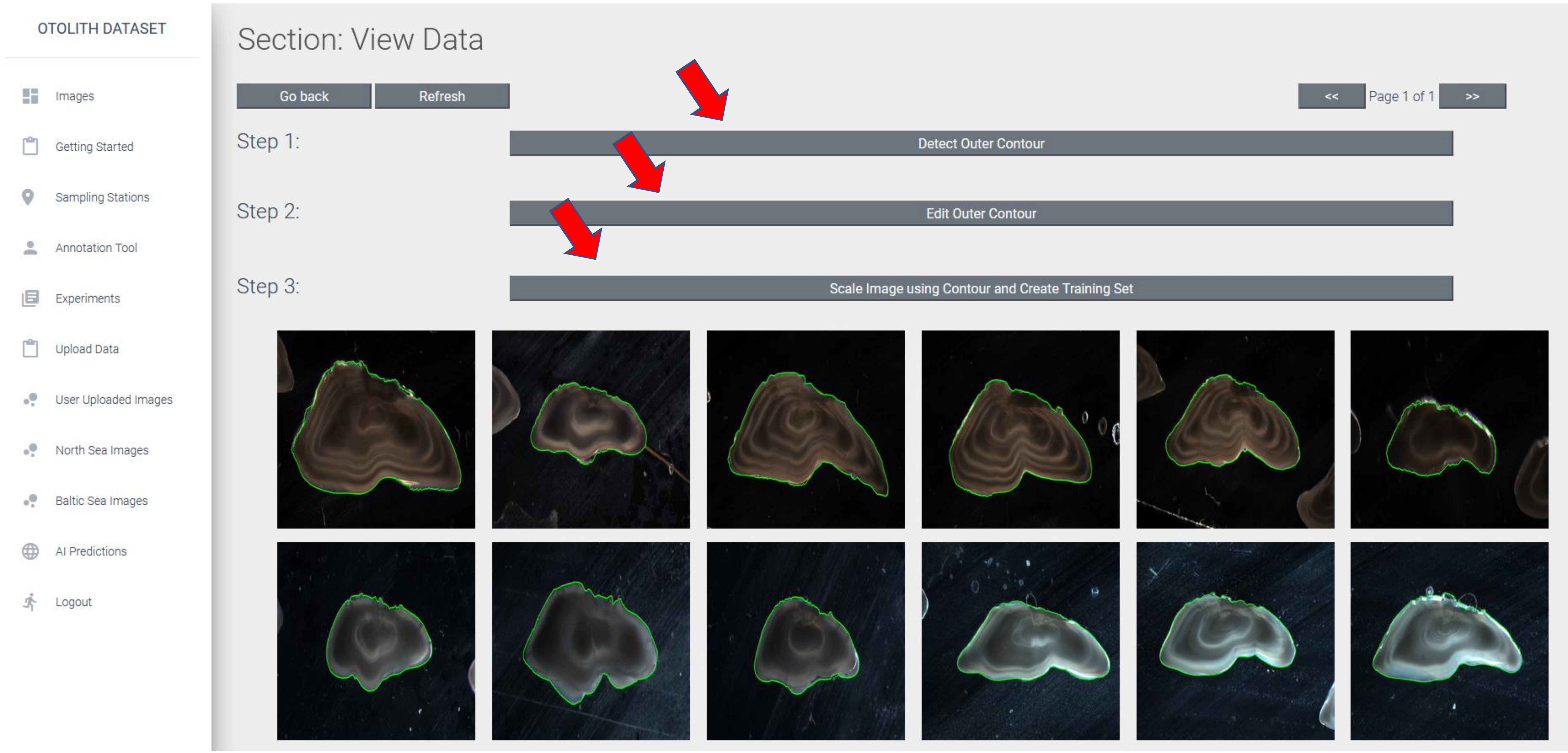
OTOLITH DATASET
Images
Getting Started
Sampling Stations
Annotation Tool
Experiments
Upload Data
User Uploaded Images
North Sea Images
Baltic Sea Images
AI Predictions
Logout
Section: View Data
Go back
Refresh
<<
Page 1 of 1
>>
Step 1:
Detect Outer Contour
Step 2:
Edit Outer Contour
Step 3:
Scale Image using Contour and Create Training Set

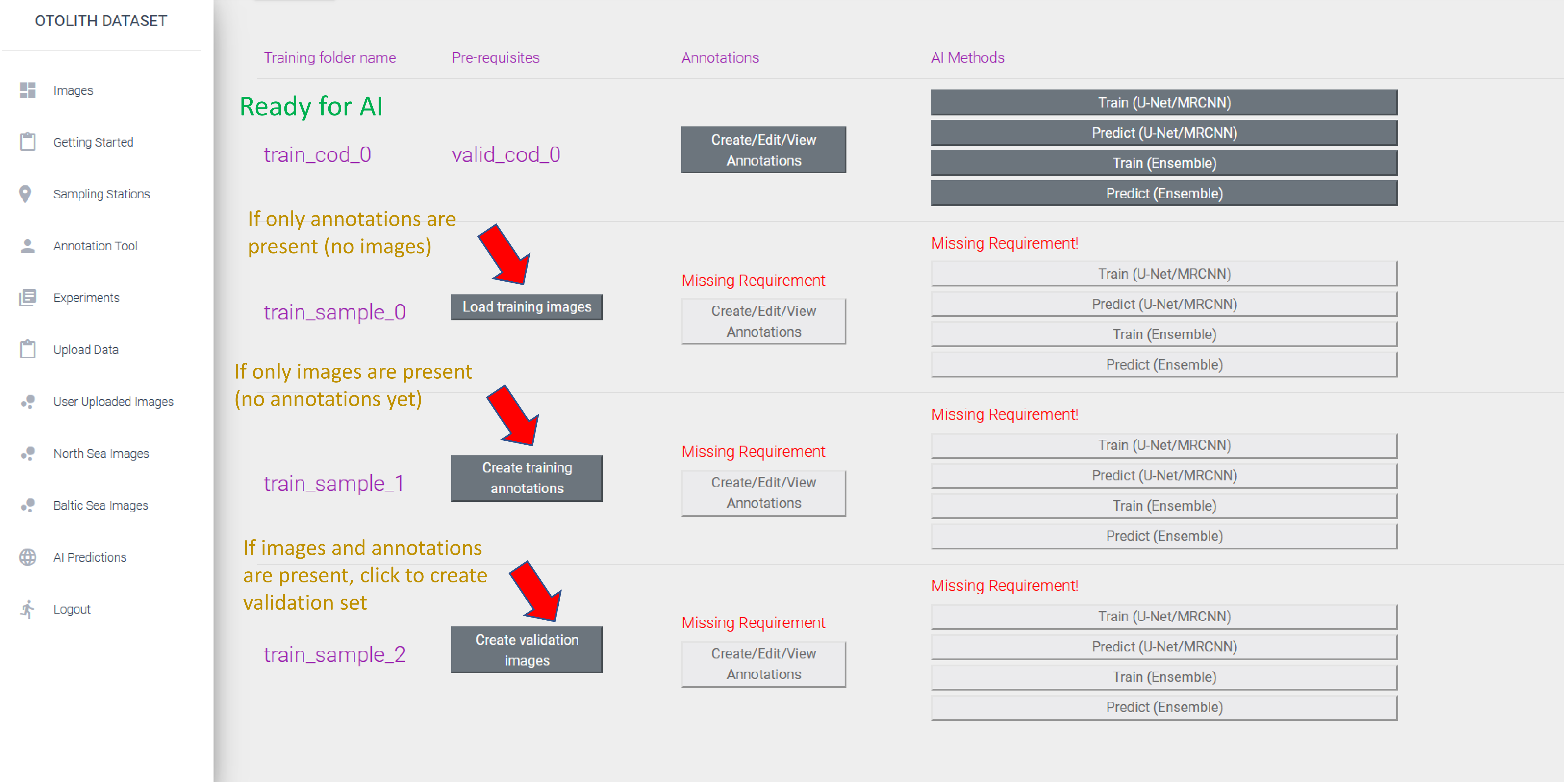
OTOLITH DATASET
Images
Getting Started
Sampling Stations
Annotation Tool
Experiments
Upload Data
User Uploaded Images
North Sea Images
Baltic Sea Images
AI Predictions
Logout
Training folder name
Pre-requisites
Annotations
AI Methods
Ready for AI
train_cod_0
valid_cod_0
Create/Edit/View Annotations
Train (U-Net/MRCNN)
Predict (U-Net/MRCNN)
Train (Ensemble)
Predict (Ensemble)
If only annotations are present (no images)
train_sample_0
Load training images
Missing Requirement
Create/Edit/View Annotations
Missing Requirement!
Train (U-Net/MRCNN)
Predict (U-Net/MRCNN)
Train (Ensemble)
Predict (Ensemble)
If only images are present (no annotations yet)
train_sample_1
Create training annotations
Missing Requirement
Create/Edit/View Annotations
Missing Requirement!
Train (U-Net/MRCNN)
Predict (U-Net/MRCNN)
Train (Ensemble)
Predict (Ensemble)
If images and annotations are present, click to create validation set
train_sample_2
Create validation images
Missing Requirement
Create/Edit/View Annotations
Missing Requirement!
Train (U-Net/MRCNN)
Predict (U-Net/MRCNN)
Train (Ensemble)
Predict (Ensemble)

Page for creating manual annotations

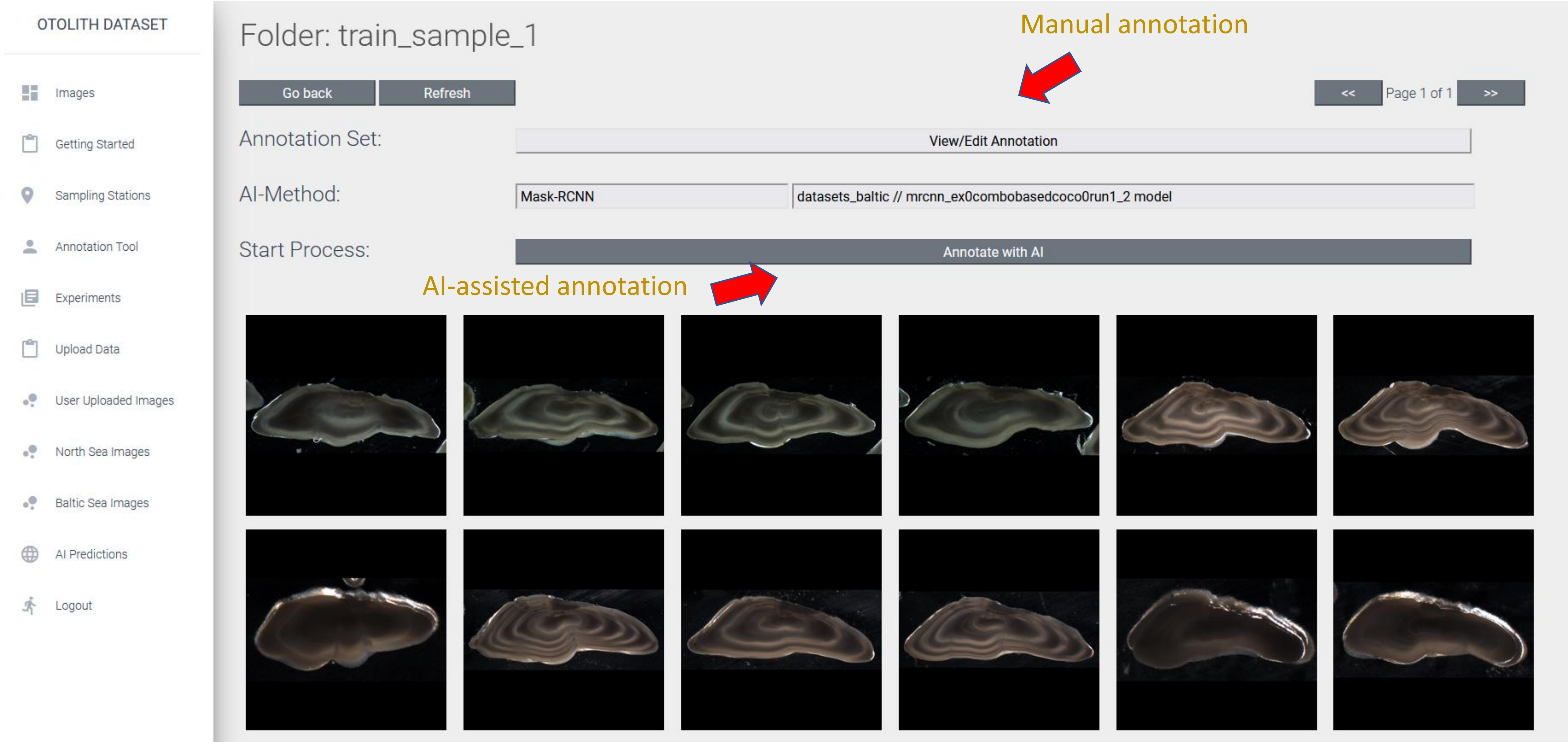

When all data requirements are completed

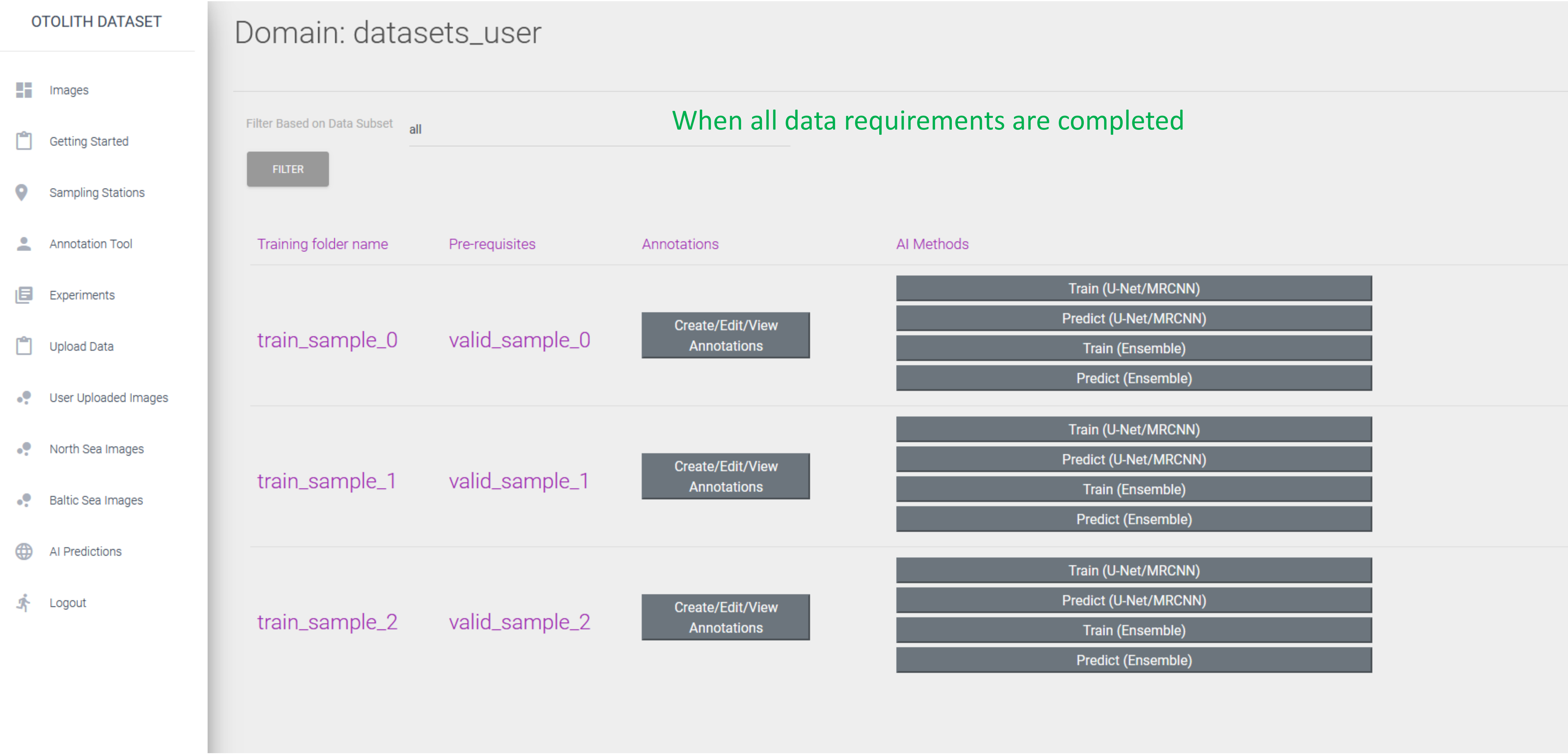

Supplement: S1 File — (ZIP) [file pone.0313934.s003.zip › USER_MANUAL/UserManual_DataPreparation.pdf]
